# Supplementary material for: Thermal Comfort Conditions and Mortality in Brazil
Source: Int J Environ Res Public Health. 2024 Sep 20;21(9):1248. doi: 10.3390/ijerph21091248 (PMC11431699; doi:10.3390/ijerph21091248)
Supplement: Supplementary file 1 [file ijerph-21-01248-s001.zip › ijerph-3202112-supplementary.pdf]

SUPPLEMENTARY MATERIALS

Thermal comfort conditions and mortality in Brazil

Weeberb J. Requia
(Corresponding Author)
Center for Environment and Public Health Studies,
School of Public Policy and Government, Fundação Getúlio Vargas
Brasília, Distrito Federal, Brazil

Reizane Maria Damasceno da Silva
Center for Environment and Public Health Studies,
School of Public Policy and Government, Fundação Getúlio Vargas
Brasília, Distrito Federal, Brazil

Leonardo Hoinaski
Federal University of Santa Catarina
Santa Catarina, Brazil

Heresh Amini
Icahn School of Medicine at Mount Sinai
New York, United States

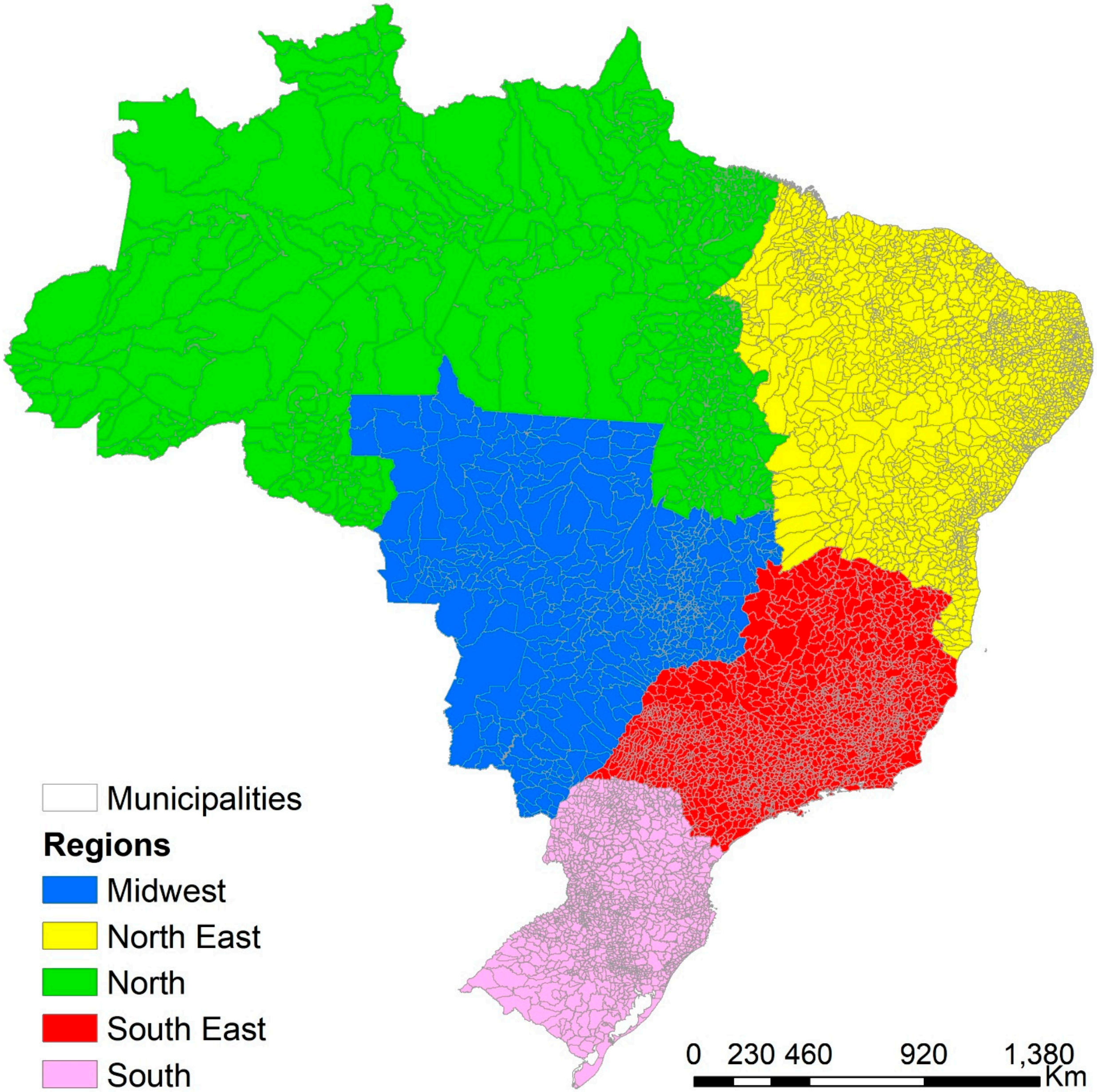

Figure S1: Spatial distribution of the municipalities and regions in Brazil.

Thermal indices

1. Discomfort Index

The Discomfort Index (DI) relies on a linear equation utilizing average air temperature and relative humidity to characterize outdoor human comfort [1]. Equation 1 delineates the DI:

$$DI = (T - 0.55) \times [(1 - 0.01) \times RH] \times (T - 14.5) \quad (1)$$

where T represents the average daily air temperature (ºC) and RH denotes relative humidity (%). In tropical environments, DI values below 15 and above 26.5 are deemed uncomfortable, while values between 15 and 20 are considered comfortable, and values between 20 and 26.5 are slightly uncomfortable [2].

2. Net Effective Temperature

Originally developed in 1937 (Missenard,1937), the Net Effective Temperature (NET) was refined by Landsberg (1972) by incorporating wind speed as a factor to calculate outdoor thermal comfort. The NET is applicable for both hot and cold conditions. Equation 2 describes the NET:

$$NET = 37 - \frac{37 - T}{[(0.68 - 0.0014) \times RH] + \frac{1}{1.76 + 1.4V^{0.75}}} - (0.29 \times T) \times [1 - (0.01 \times RH)] \quad (2)$$

where T represents the average daily air temperature (ºC), RH represents relative humidity (%), and V represents wind speed (m/s). NET values ranging from 17 to 21 are considered comfortable. Conversely, values between 9 and 17 indicate mild discomfort in cold conditions, while those falling between 1 and 9 suggest cold temperatures, and values below 1 are considered very cold. On the other hand, NET values ranging from 21 to 23 are categorized as slightly hot, those between 23 and 27 as hot, and values exceeding 27 as very hot.

3. Humidex

Humidex (H) has been extensively used in thermal studies, encompassing both urban and rural areas [3] Developed by the Atmospheric Environment Service from Canada (Masterton and Richardson, 1979), this index incorporates air vapor pressure in the calculation, as illustrated in Equations 3 and 4:

$$Pas = 6.112 \times \left(10 \times \frac{7.5 \times T}{237.7 \times T}\right) \times \frac{RH}{100} \tag{3}$$

$$H = T + \frac{5}{9} \times (Pas - 10) \tag{4}$$

where Pas denotes air vapor pressure. Humidex values below 29 indicate no discomfort, while values between 30 and 39 suggest mild discomfort, those falling between 40 and 45 indicate significant discomfort, and values exceeding 45 indicate a dangerous level of discomfort.

4. Heat Index

The Heat Index (HI) is utilized by the National Oceanic and Atmospheric Administration (NOAA). Derived from regression analysis considering the influence of air temperature and relative humidity on human thermal comfort [4]. Equation 5 outlines the HI:

$$\begin{aligned} HI = -8.784695 &+ (1.61139411 \times T) + (2.338549 \times RH) - (0.14611605 \times T \times RH) \\ &- (1.2308094 \times 10^{-2} \times T^2) - (1.6424828 \times 10^{-2} \times RH^2) \\ &+ (2.211732 \times 10^{-3} \times T^2 \times RH) + (7.2546 \times 10^{-4} \times T \times RH^2) \\ &- 3.582 \times 10^{-6} \times T^2 \times RH^2 \end{aligned} \tag{5}$$

For the HI, values below 27 are considered comfortable, those ranging from 27 to 32 indicate caution, while values above 32 represent the onset of a dangerous situation [4].

References:

1. Thom, E.C., The Discomfort Index. *Weatherwise* **1959** *12*, 57–61. https://doi.org/10.1080/00431672.1959.9926960
2. Md Din, M.F., Lee, Y.Y., Ponraj, M., Ossen, D.R., Iwao, K., Chelliapan, S., Thermal comfort of various building layouts with a proposed discomfort index range for tropical climate. *J Therm Biol* **2014**, *41*, 6–15. https://doi.org/10.1016/J.JTHERBIO.2014.01.004
3. Coccolo, S.; Kämpf, J.; Scartezzini, J. L.; Pearlmutter, D. Outdoor Human Comfort and Thermal Stress: A Comprehensive Review on Models and Standards. *Urban Clim* **2016**, *18*, 33–57. https://doi.org/10.1016/J.UCLIM.2016.08.004.
4. Rothfusz, L.P. *The Heat Index Equation*. National Weather Service Technical Attachment (SR 90-23): College Park, MD, USA 1990

Table S1 - National average RR (from the primary analysis, sensitivity analyses, and all sub-group analyses) along with the estimated heterogeneity test from the meta-analysis.

Outcome	Group	Lag	Index	national_RR_LOWEST	Lower95%CI_LOWEST	Upper95%CI_LOWEST	national_RR_HIGHEST	Lower95%CI_HIGHEST	Upper95%CI_HIGHEST
Circulatory	allAges_allSex	3	temp_index_1	1.152	1.103	1.200	1.081	1.026	1.135
Circulatory	allAges_allSex	3	temp_index_2	1.121	1.083	1.158	1.063	1.025	1.100
Circulatory	allAges_allSex	3	temp_index_3	1.147	1.098	1.197	1.059	0.993	1.125
Circulatory	allAges_allSex	3	temp_index_4	1.137	1.094	1.181	1.057	1.015	1.098
Circulatory	allAges_allSex	5	temp_index_1	1.168	1.112	1.223	1.085	1.024	1.147
Circulatory	allAges_allSex	5	temp_index_2	1.144	1.099	1.189	1.060	1.019	1.101
Circulatory	allAges_allSex	5	temp_index_3	1.162	1.105	1.219	1.059	0.985	1.133
Circulatory	allAges_allSex	5	temp_index_4	1.154	1.104	1.204	1.066	1.018	1.114
Circulatory	allAges_allSex	7	temp_index_1	1.182	1.119	1.246	1.055	0.986	1.123
Circulatory	allAges_allSex	7	temp_index_2	1.165	1.122	1.207	1.047	0.979	1.114
Circulatory	allAges_allSex	7	temp_index_3	1.181	1.115	1.248	1.052	0.967	1.137
Circulatory	allAges_allSex	7	temp_index_4	1.193	1.148	1.237	1.063	0.973	1.154
Circulatory	allAges_allSex	10	temp_index_1	1.258	1.199	1.318	1.034	0.917	1.152
Circulatory	allAges_allSex	10	temp_index_2	1.189	1.136	1.241	1.020	0.944	1.097
Circulatory	allAges_allSex	10	temp_index_3	1.262	1.197	1.327	1.033	0.868	1.198
Circulatory	allAges_allSex	10	temp_index_4	1.242	1.186	1.297	1.058	0.952	1.164
Circulatory	allAges_allSex	15	temp_index_1	1.326	1.248	1.404	1.007	0.858	1.157
Circulatory	allAges_allSex	15	temp_index_2	1.234	1.165	1.304	1.010	0.911	1.108
Circulatory	allAges_allSex	15	temp_index_3	1.352	1.265	1.439	0.977	0.757	1.197
Circulatory	allAges_allSex	15	temp_index_4	1.313	1.239	1.386	1.052	0.914	1.191
Circulatory	allAges_allSex	20	temp_index_1	1.297	1.182	1.413	1.020	0.894	1.146
Circulatory	allAges_allSex	20	temp_index_2	1.223	1.133	1.314	0.962	0.878	1.046
Circulatory	allAges_allSex	20	temp_index_3	1.316	1.195	1.438	0.985	0.816	1.154
Circulatory	allAges_allSex	20	temp_index_4	1.289	1.180	1.397	1.045	0.938	1.151
Circulatory	allAges_Men	3	temp_index_1	1.189	1.113	1.266	1.046	0.973	1.118
Circulatory	allAges_Men	3	temp_index_2	1.135	1.084	1.186	1.034	0.985	1.084
Circulatory	allAges_Men	3	temp_index_3	1.187	1.105	1.268	1.014	0.925	1.103
Circulatory	allAges_Men	3	temp_index_4	1.132	1.060	1.203	1.018	0.963	1.072
Circulatory	allAges_Men	5	temp_index_1	1.209	1.123	1.295	1.050	0.968	1.131
Circulatory	allAges_Men	5	temp_index_2	1.149	1.089	1.208	1.015	0.961	1.069
Circulatory	allAges_Men	5	temp_index_3	1.204	1.113	1.295	1.014	0.914	1.114
Circulatory	allAges_Men	5	temp_index_4	1.182	1.113	1.251	1.040	0.975	1.106
Circulatory	allAges_Men	7	temp_index_1	1.222	1.124	1.319	1.008	0.918	1.099

Circulatory	allAges_Men	7	temp_index_2	1.175	1.119	1.230	0.990	0.903	1.077
Circulatory	allAges_Men	7	temp_index_3	1.221	1.118	1.324	0.980	0.870	1.090
Circulatory	allAges_Men	7	temp_index_4	1.193	1.129	1.257	0.932	0.826	1.037
Circulatory	allAges_Men	10	temp_index_1	1.300	1.177	1.424	1.006	0.901	1.111
Circulatory	allAges_Men	10	temp_index_2	1.196	1.122	1.270	0.964	0.870	1.057
Circulatory	allAges_Men	10	temp_index_3	1.306	1.176	1.435	0.978	0.849	1.107
Circulatory	allAges_Men	10	temp_index_4	1.248	1.167	1.329	0.943	0.819	1.067
Circulatory	allAges_Men	15	temp_index_1	1.415	1.245	1.584	0.990	0.854	1.126
Circulatory	allAges_Men	15	temp_index_2	1.275	1.153	1.397	0.921	0.841	1.001
Circulatory	allAges_Men	15	temp_index_3	1.429	1.254	1.603	0.949	0.761	1.138
Circulatory	allAges_Men	15	temp_index_4	1.354	1.219	1.489	0.996	0.888	1.105
Circulatory	allAges_Men	20	temp_index_1	1.437	1.251	1.622	1.033	0.857	1.209
Circulatory	allAges_Men	20	temp_index_2	1.290	1.144	1.436	0.929	0.830	1.027
Circulatory	allAges_Men	20	temp_index_3	1.433	1.241	1.624	0.978	0.764	1.193
Circulatory	allAges_Men	20	temp_index_4	1.391	1.226	1.557	1.019	0.881	1.158
Circulatory	allAges_Women	3	temp_index_1	1.105	1.035	1.175	1.110	1.028	1.192
Circulatory	allAges_Women	3	temp_index_2	1.112	1.069	1.154	1.141	1.054	1.227
Circulatory	allAges_Women	3	temp_index_3	1.106	1.035	1.177	1.090	0.992	1.189
Circulatory	allAges_Women	3	temp_index_4	1.103	1.040	1.166	1.090	1.027	1.153
Circulatory	allAges_Women	5	temp_index_1	1.112	1.031	1.194	1.121	1.027	1.215
Circulatory	allAges_Women	5	temp_index_2	1.115	1.048	1.182	1.103	1.040	1.166
Circulatory	allAges_Women	5	temp_index_3	1.122	1.039	1.205	1.102	0.988	1.216
Circulatory	allAges_Women	5	temp_index_4	1.121	1.047	1.195	1.096	1.024	1.168
Circulatory	allAges_Women	7	temp_index_1	1.131	1.037	1.225	1.103	0.996	1.210
Circulatory	allAges_Women	7	temp_index_2	1.124	1.046	1.203	1.079	1.008	1.149
Circulatory	allAges_Women	7	temp_index_3	1.153	1.051	1.254	1.116	0.978	1.253
Circulatory	allAges_Women	7	temp_index_4	1.135	1.049	1.221	1.084	0.999	1.169
Circulatory	allAges_Women	10	temp_index_1	1.234	1.149	1.318	1.161	0.947	1.375
Circulatory	allAges_Women	10	temp_index_2	1.178	1.098	1.257	1.111	0.982	1.240
Circulatory	allAges_Women	10	temp_index_3	1.260	1.170	1.351	1.265	0.911	1.618
Circulatory	allAges_Women	10	temp_index_4	1.218	1.136	1.299	1.193	1.002	1.384
Circulatory	allAges_Women	15	temp_index_1	1.275	1.164	1.387	1.086	0.820	1.353
Circulatory	allAges_Women	15	temp_index_2	1.209	1.106	1.312	1.143	0.965	1.321
Circulatory	allAges_Women	15	temp_index_3	1.314	1.189	1.439	1.057	0.654	1.460
Circulatory	allAges_Women	15	temp_index_4	1.251	1.144	1.358	1.159	0.915	1.403
Circulatory	allAges_Women	20	temp_index_1	1.276	1.140	1.412	0.983	0.685	1.281
Circulatory	allAges_Women	20	temp_index_2	1.188	1.070	1.307	1.052	0.855	1.250
Circulatory	allAges_Women	20	temp_index_3	1.301	1.152	1.449	0.924	0.481	1.366
Circulatory	allAges_Women	20	temp_index_4	1.240	1.117	1.363	1.046	0.774	1.317

Circulatory	Age0_14_allSex	3	temp_index_1	0.667	0.314	1.021	0.237	-0.120	0.594
Circulatory	Age0_14_allSex	3	temp_index_2	0.844	0.440	1.247	0.638	-0.025	1.301
Circulatory	Age0_14_allSex	3	temp_index_3	0.804	0.263	1.344	0.535	-0.626	1.696
Circulatory	Age0_14_allSex	3	temp_index_4	0.647	0.350	0.945	0.431	-0.239	1.100
Circulatory	Age0_14_allSex	5	temp_index_1	0.803	0.224	1.382	0.257	-0.217	0.730
Circulatory	Age0_14_allSex	5	temp_index_2	0.710	0.628	0.792	0.694	0.091	1.296
Circulatory	Age0_14_allSex	5	temp_index_3	1.214	0.529	1.898	0.259	-0.595	1.112
Circulatory	Age0_14_allSex	5	temp_index_4	0.820	0.291	1.348	0.597	-0.362	1.557
Circulatory	Age0_14_allSex	7	temp_index_1	1.014	0.072	1.955	0.214	-0.144	0.571
Circulatory	Age0_14_allSex	7	temp_index_2	0.942	-0.091	1.976	0.660	-0.069	1.388
Circulatory	Age0_14_allSex	7	temp_index_3	1.189	0.283	2.094	-0.053	-0.424	0.318
Circulatory	Age0_14_allSex	7	temp_index_4	1.183	0.091	2.275	0.747	-0.756	2.251
Circulatory	Age0_14_allSex	10	temp_index_1	0.742	0.346	1.137	0.212	-0.103	0.527
Circulatory	Age0_14_allSex	10	temp_index_2	1.058	-0.106	2.222	0.532	-0.387	1.451
Circulatory	Age0_14_allSex	10	temp_index_3	0.798	-0.049	1.644	-0.007	-0.282	0.269
Circulatory	Age0_14_allSex	10	temp_index_4	1.210	0.202	2.218	0.245	-0.153	0.643
Circulatory	Age0_14_allSex	15	temp_index_1	0.780	0.218	1.341	2.03619677059928e-09	0.000	4.52242760510666e-05
Circulatory	Age0_14_allSex	15	temp_index_2	0.621	0.322	0.921	0.170	-0.202	0.542
Circulatory	Age0_14_allSex	15	temp_index_3	0.708	0.117	1.298	1.50817808584171e-09	0.000	4.5223748101329e-05
Circulatory	Age0_14_allSex	15	temp_index_4	0.755	0.305	1.205	0.000	-0.011	0.011
Circulatory	Age0_14_allSex	20	temp_index_1	0.954	-0.112	2.021	1.41973341610302e-09	0.000	4.52236596344981e-05
Circulatory	Age0_14_allSex	20	temp_index_2	0.819	0.296	1.342	0.013	-0.340	0.365
Circulatory	Age0_14_allSex	20	temp_index_3	0.477	0.331	0.623	3.07090584830532e-09	0.000	4.52253104445278e-05
Circulatory	Age0_14_allSex	20	temp_index_4	0.703	0.098	1.307	1.88638819580927e-09	0.000	4.52241262493379e-05
Circulatory	Age15_45_allSex	3	temp_index_1	0.880	0.766	0.993	1.088	0.887	1.289
Circulatory	Age15_45_allSex	3	temp_index_2	1.180	1.028	1.332	1.030	0.901	1.159
Circulatory	Age15_45_allSex	3	temp_index_3	0.868	0.749	0.986	1.092	0.839	1.344
Circulatory	Age15_45_allSex	3	temp_index_4	1.153	1.015	1.290	0.953	0.828	1.077
Circulatory	Age15_45_allSex	5	temp_index_1	0.994	0.848	1.140	1.065	0.841	1.289
Circulatory	Age15_45_allSex	5	temp_index_2	1.232	1.046	1.418	0.971	0.841	1.101
Circulatory	Age15_45_allSex	5	temp_index_3	0.962	0.812	1.112	1.042	0.767	1.316
Circulatory	Age15_45_allSex	5	temp_index_4	1.255	1.052	1.459	0.937	0.801	1.072
Circulatory	Age15_45_allSex	7	temp_index_1	1.277	1.030	1.524	0.988	0.747	1.229
Circulatory	Age15_45_allSex	7	temp_index_2	1.263	1.049	1.477	0.945	0.801	1.088
Circulatory	Age15_45_allSex	7	temp_index_3	1.257	1.009	1.505	0.989	0.551	1.427
Circulatory	Age15_45_allSex	7	temp_index_4	1.258	1.043	1.474	0.918	0.761	1.074
Circulatory	Age15_45_allSex	10	temp_index_1	1.243	0.962	1.525	0.979	0.692	1.266
Circulatory	Age15_45_allSex	10	temp_index_2	1.245	1.031	1.459	1.057	0.735	1.379
Circulatory	Age15_45_allSex	10	temp_index_3	1.230	1.008	1.453	1.792	0.189	3.395

Circulatory	Age15_45_allSex	10	temp_index_4	1.234	0.984	1.484	0.968	0.744	1.192
Circulatory	Age15_45_allSex	15	temp_index_1	1.026	0.782	1.269	0.995	0.603	1.388
Circulatory	Age15_45_allSex	15	temp_index_2	1.235	0.963	1.507	1.039	0.623	1.455
Circulatory	Age15_45_allSex	15	temp_index_3	1.034	0.758	1.310	0.968	0.452	1.485
Circulatory	Age15_45_allSex	15	temp_index_4	1.060	0.820	1.300	0.883	0.618	1.148
Circulatory	Age15_45_allSex	20	temp_index_1	0.933	0.666	1.201	1.076	0.521	1.631
Circulatory	Age15_45_allSex	20	temp_index_2	1.252	0.931	1.573	1.198	0.587	1.808
Circulatory	Age15_45_allSex	20	temp_index_3	0.921	0.630	1.212	0.301	0.093	0.509
Circulatory	Age15_45_allSex	20	temp_index_4	1.036	0.753	1.319	0.868	0.572	1.164
Circulatory	Age46_65_allSex	3	temp_index_1	1.165	1.062	1.267	0.965	0.874	1.056
Circulatory	Age46_65_allSex	3	temp_index_2	1.167	1.078	1.255	0.983	0.919	1.046
Circulatory	Age46_65_allSex	3	temp_index_3	1.199	1.111	1.288	0.910	0.739	1.081
Circulatory	Age46_65_allSex	3	temp_index_4	1.129	1.046	1.213	0.946	0.850	1.041
Circulatory	Age46_65_allSex	5	temp_index_1	1.153	1.046	1.260	0.929	0.837	1.021
Circulatory	Age46_65_allSex	5	temp_index_2	1.142	1.044	1.241	0.942	0.874	1.010
Circulatory	Age46_65_allSex	5	temp_index_3	1.210	1.108	1.312	0.800	0.631	0.969
Circulatory	Age46_65_allSex	5	temp_index_4	1.096	0.994	1.198	0.950	0.872	1.028
Circulatory	Age46_65_allSex	7	temp_index_1	1.207	1.092	1.322	0.820	0.692	0.948
Circulatory	Age46_65_allSex	7	temp_index_2	1.156	1.042	1.271	0.884	0.813	0.956
Circulatory	Age46_65_allSex	7	temp_index_3	1.252	1.111	1.394	0.829	0.682	0.976
Circulatory	Age46_65_allSex	7	temp_index_4	1.156	1.044	1.267	0.827	0.714	0.941
Circulatory	Age46_65_allSex	10	temp_index_1	1.330	1.184	1.475	0.870	0.696	1.044
Circulatory	Age46_65_allSex	10	temp_index_2	1.216	1.073	1.360	0.878	0.797	0.960
Circulatory	Age46_65_allSex	10	temp_index_3	1.357	1.189	1.525	0.863	0.623	1.102
Circulatory	Age46_65_allSex	10	temp_index_4	1.281	1.134	1.429	0.857	0.711	1.002
Circulatory	Age46_65_allSex	15	temp_index_1	1.355	1.203	1.507	0.827	0.581	1.074
Circulatory	Age46_65_allSex	15	temp_index_2	1.267	1.101	1.433	0.868	0.752	0.983
Circulatory	Age46_65_allSex	15	temp_index_3	1.397	1.225	1.569	0.755	0.430	1.081
Circulatory	Age46_65_allSex	15	temp_index_4	1.316	1.145	1.487	0.880	0.655	1.104
Circulatory	Age46_65_allSex	20	temp_index_1	1.298	1.133	1.463	0.743	0.468	1.017
Circulatory	Age46_65_allSex	20	temp_index_2	1.223	1.022	1.423	0.807	0.695	0.918
Circulatory	Age46_65_allSex	20	temp_index_3	1.350	1.154	1.547	0.696	0.319	1.073
Circulatory	Age46_65_allSex	20	temp_index_4	1.207	1.031	1.383	0.727	0.504	0.951
Circulatory	Age65_allSex	3	temp_index_1	1.150	1.091	1.208	1.138	1.065	1.210
Circulatory	Age65_allSex	3	temp_index_2	1.109	1.062	1.155	1.110	1.061	1.159
Circulatory	Age65_allSex	3	temp_index_3	1.133	1.072	1.194	1.105	1.017	1.192
Circulatory	Age65_allSex	3	temp_index_4	1.144	1.091	1.198	1.109	1.050	1.168
Circulatory	Age65_allSex	5	temp_index_1	1.163	1.096	1.230	1.157	1.074	1.240
Circulatory	Age65_allSex	5	temp_index_2	1.133	1.095	1.171	1.166	1.081	1.251

Circulatory	Age65_allSex	5	temp_index_3	1.149	1.080	1.219	1.121	1.021	1.221
Circulatory	Age65_allSex	5	temp_index_4	1.160	1.099	1.222	1.131	1.060	1.201
Circulatory	Age65_allSex	7	temp_index_1	1.172	1.096	1.249	1.139	1.047	1.232
Circulatory	Age65_allSex	7	temp_index_2	1.148	1.099	1.196	1.151	1.055	1.247
Circulatory	Age65_allSex	7	temp_index_3	1.157	1.081	1.233	1.141	1.014	1.269
Circulatory	Age65_allSex	7	temp_index_4	1.198	1.146	1.249	1.173	1.044	1.302
Circulatory	Age65_allSex	10	temp_index_1	1.248	1.177	1.318	1.098	0.939	1.257
Circulatory	Age65_allSex	10	temp_index_2	1.175	1.112	1.238	1.127	1.018	1.235
Circulatory	Age65_allSex	10	temp_index_3	1.245	1.172	1.318	1.053	0.831	1.274
Circulatory	Age65_allSex	10	temp_index_4	1.236	1.170	1.302	1.149	0.999	1.298
Circulatory	Age65_allSex	15	temp_index_1	1.347	1.250	1.444	1.056	0.861	1.252
Circulatory	Age65_allSex	15	temp_index_2	1.234	1.149	1.320	1.094	0.958	1.231
Circulatory	Age65_allSex	15	temp_index_3	1.362	1.253	1.470	0.962	0.688	1.235
Circulatory	Age65_allSex	15	temp_index_4	1.330	1.236	1.423	1.111	0.928	1.294
Circulatory	Age65_allSex	20	temp_index_1	1.337	1.190	1.485	1.107	0.936	1.278
Circulatory	Age65_allSex	20	temp_index_2	1.261	1.159	1.364	1.069	0.911	1.228
Circulatory	Age65_allSex	20	temp_index_3	1.351	1.184	1.518	1.041	0.813	1.270
Circulatory	Age65_allSex	20	temp_index_4	1.327	1.188	1.467	1.149	0.990	1.308
Circulatory	Age0_14_Men	3	temp_index_1	1.007	0.531	1.483	0.158	-0.520	0.835
Circulatory	Age0_14_Men	3	temp_index_2	0.985	0.698	1.272	0.245	-0.112	0.603
Circulatory	Age0_14_Men	3	temp_index_3	1.552	-0.392	3.496	0.193	-0.355	0.742
Circulatory	Age0_14_Men	3	temp_index_4	0.972	0.711	1.232	0.180	-0.095	0.455
Circulatory	Age0_14_Men	5	temp_index_1	0.507	-0.149	1.163	0.243	-1.250	1.736
Circulatory	Age0_14_Men	5	temp_index_2	0.989	0.661	1.316	0.183	-0.120	0.486
Circulatory	Age0_14_Men	5	temp_index_3	4.535	3.252	5.818	0.001	-0.013	0.015
Circulatory	Age0_14_Men	5	temp_index_4	0.786	0.341	1.231	0.229	-0.130	0.588
Circulatory	Age0_14_Men	7	temp_index_1	0.666	-0.855	2.187	0.131	-0.883	1.144
Circulatory	Age0_14_Men	7	temp_index_2	0.342	0.063	0.621	0.187	-0.320	0.695
Circulatory	Age0_14_Men	7	temp_index_3	0.383	0.256	0.510	-0.043	-0.441	0.356
Circulatory	Age0_14_Men	7	temp_index_4	0.556	-0.118	1.230	0.220	-0.179	0.620
Circulatory	Age0_14_Men	10	temp_index_1	0.254	0.189	0.319	0.184	-0.641	1.008
Circulatory	Age0_14_Men	10	temp_index_2	0.738	-0.070	1.546	0.119	-0.165	0.402
Circulatory	Age0_14_Men	10	temp_index_3	0.378	0.245	0.512	-0.150	-1.015	0.715
Circulatory	Age0_14_Men	10	temp_index_4	0.226	0.157	0.295	0.223	-0.250	0.696
Circulatory	Age0_14_Men	15	temp_index_1	0.136	-0.012	0.285	0.038	-0.084	0.160
Circulatory	Age0_14_Men	15	temp_index_2	0.124	-0.011	0.259	0.092	-0.416	0.600
Circulatory	Age0_14_Men	15	temp_index_3	0.480	0.314	0.647	2.4771671761231e-09	0.000	4.52247164519983e-05
Circulatory	Age0_14_Men	15	temp_index_4	1.159	-0.764	3.083	4.42218739818442e-09	0.000	4.52266602178776e-05
Circulatory	Age0_14_Men	20	temp_index_1	0.147	-0.047	0.341	2.83091797366824e-09	0.000	4.52250697309367e-05

Circulatory	Age0_14_Men	20	temp_index_2	0.336	0.097	0.574	-0.006	-0.375	0.362
Circulatory	Age0_14_Men	20	temp_index_3	0.527	0.326	0.729	1.93571055461128e-09	0.000	4.52241750278341e-05
Circulatory	Age0_14_Men	20	temp_index_4	0.308	0.063	0.553	3.14387090137482e-09	0.000	4.52253827083132e-05
Circulatory	Age15_45_Men	3	temp_index_1	0.997	0.834	1.161	1.061	0.789	1.333
Circulatory	Age15_45_Men	3	temp_index_2	1.346	1.097	1.596	1.010	0.849	1.170
Circulatory	Age15_45_Men	3	temp_index_3	0.880	0.711	1.049	1.044	0.703	1.385
Circulatory	Age15_45_Men	3	temp_index_4	1.000	0.850	1.151	0.890	0.743	1.036
Circulatory	Age15_45_Men	5	temp_index_1	1.132	0.916	1.348	1.027	0.736	1.319
Circulatory	Age15_45_Men	5	temp_index_2	1.388	1.110	1.667	0.947	0.786	1.109
Circulatory	Age15_45_Men	5	temp_index_3	1.055	0.852	1.258	0.993	0.630	1.355
Circulatory	Age15_45_Men	5	temp_index_4	1.133	0.933	1.333	0.851	0.701	1.001
Circulatory	Age15_45_Men	7	temp_index_1	1.234	0.968	1.500	0.963	0.648	1.279
Circulatory	Age15_45_Men	7	temp_index_2	1.478	1.116	1.840	0.928	0.745	1.111
Circulatory	Age15_45_Men	7	temp_index_3	1.363	1.070	1.655	0.605	0.328	0.883
Circulatory	Age15_45_Men	7	temp_index_4	1.186	0.953	1.420	0.838	0.668	1.008
Circulatory	Age15_45_Men	10	temp_index_1	1.145	0.858	1.432	1.056	0.633	1.479
Circulatory	Age15_45_Men	10	temp_index_2	1.343	0.978	1.708	0.937	0.696	1.178
Circulatory	Age15_45_Men	10	temp_index_3	1.038	0.747	1.330	1.068	0.502	1.634
Circulatory	Age15_45_Men	10	temp_index_4	1.108	0.853	1.364	0.880	0.636	1.123
Circulatory	Age15_45_Men	15	temp_index_1	1.048	0.709	1.386	1.108	0.502	1.713
Circulatory	Age15_45_Men	15	temp_index_2	1.336	0.864	1.808	0.907	0.605	1.208
Circulatory	Age15_45_Men	15	temp_index_3	0.991	0.649	1.334	1.138	0.180	2.097
Circulatory	Age15_45_Men	15	temp_index_4	0.951	0.658	1.245	0.801	0.512	1.090
Circulatory	Age15_45_Men	20	temp_index_1	1.076	0.600	1.551	1.182	0.325	2.039
Circulatory	Age15_45_Men	20	temp_index_2	1.306	0.872	1.741	1.017	0.254	1.780
Circulatory	Age15_45_Men	20	temp_index_3	0.988	0.529	1.446	0.334	-0.198	0.865
Circulatory	Age15_45_Men	20	temp_index_4	0.953	0.558	1.348	0.764	0.432	1.095
Circulatory	Age46_65_Men	3	temp_index_1	1.109	0.983	1.234	0.926	0.810	1.043
Circulatory	Age46_65_Men	3	temp_index_2	1.106	0.998	1.215	0.954	0.872	1.035
Circulatory	Age46_65_Men	3	temp_index_3	1.119	0.985	1.252	0.904	0.760	1.048
Circulatory	Age46_65_Men	3	temp_index_4	1.101	0.995	1.206	0.898	0.776	1.020
Circulatory	Age46_65_Men	5	temp_index_1	1.128	1.013	1.243	0.825	0.664	0.985
Circulatory	Age46_65_Men	5	temp_index_2	1.109	0.987	1.232	0.901	0.815	0.987
Circulatory	Age46_65_Men	5	temp_index_3	1.150	1.023	1.277	0.744	0.543	0.944
Circulatory	Age46_65_Men	5	temp_index_4	1.117	1.008	1.226	0.836	0.695	0.977
Circulatory	Age46_65_Men	7	temp_index_1	1.164	1.052	1.276	0.730	0.560	0.899
Circulatory	Age46_65_Men	7	temp_index_2	1.097	0.960	1.235	0.833	0.746	0.920
Circulatory	Age46_65_Men	7	temp_index_3	1.194	1.014	1.373	0.848	0.715	0.980
Circulatory	Age46_65_Men	7	temp_index_4	1.129	1.001	1.257	0.761	0.612	0.910

Circulatory	Age46_65_Men	10	temp_index_1	1.265	1.077	1.452	0.824	0.623	1.025
Circulatory	Age46_65_Men	10	temp_index_2	1.182	0.999	1.364	0.827	0.729	0.924
Circulatory	Age46_65_Men	10	temp_index_3	1.330	1.099	1.561	0.815	0.573	1.057
Circulatory	Age46_65_Men	10	temp_index_4	1.252	1.053	1.451	0.827	0.662	0.991
Circulatory	Age46_65_Men	15	temp_index_1	1.248	1.074	1.422	0.750	0.463	1.037
Circulatory	Age46_65_Men	15	temp_index_2	1.226	0.982	1.469	0.801	0.678	0.923
Circulatory	Age46_65_Men	15	temp_index_3	1.304	1.098	1.511	0.659	0.295	1.024
Circulatory	Age46_65_Men	15	temp_index_4	1.263	1.044	1.483	0.824	0.560	1.088
Circulatory	Age46_65_Men	20	temp_index_1	1.159	1.005	1.312	0.741	0.372	1.111
Circulatory	Age46_65_Men	20	temp_index_2	1.151	0.897	1.404	0.769	0.628	0.910
Circulatory	Age46_65_Men	20	temp_index_3	0.846	0.650	1.042	0.802	0.486	1.118
Circulatory	Age46_65_Men	20	temp_index_4	1.163	0.932	1.394	0.751	0.451	1.052
Circulatory	Age65_Men	3	temp_index_1	1.195	1.110	1.280	1.112	1.012	1.213
Circulatory	Age65_Men	3	temp_index_2	1.128	1.062	1.194	1.088	1.022	1.155
Circulatory	Age65_Men	3	temp_index_3	1.203	1.097	1.310	1.074	0.951	1.196
Circulatory	Age65_Men	3	temp_index_4	1.187	1.109	1.266	1.086	1.005	1.168
Circulatory	Age65_Men	5	temp_index_1	1.216	1.119	1.314	1.148	1.032	1.264
Circulatory	Age65_Men	5	temp_index_2	1.139	1.064	1.214	1.095	1.020	1.170
Circulatory	Age65_Men	5	temp_index_3	1.205	1.109	1.302	1.105	0.952	1.259
Circulatory	Age65_Men	5	temp_index_4	1.199	1.110	1.288	1.124	1.023	1.226
Circulatory	Age65_Men	7	temp_index_1	1.236	1.127	1.346	1.112	0.984	1.241
Circulatory	Age65_Men	7	temp_index_2	1.158	1.090	1.226	1.093	0.968	1.218
Circulatory	Age65_Men	7	temp_index_3	1.222	1.095	1.348	1.096	0.917	1.275
Circulatory	Age65_Men	7	temp_index_4	1.249	1.174	1.325	1.084	0.918	1.249
Circulatory	Age65_Men	10	temp_index_1	1.318	1.202	1.434	1.012	0.816	1.208
Circulatory	Age65_Men	10	temp_index_2	1.195	1.102	1.288	1.066	0.929	1.204
Circulatory	Age65_Men	10	temp_index_3	1.292	1.177	1.408	0.925	0.659	1.191
Circulatory	Age65_Men	10	temp_index_4	1.269	1.168	1.371	0.996	0.818	1.174
Circulatory	Age65_Men	15	temp_index_1	1.444	1.250	1.639	1.088	0.894	1.281
Circulatory	Age65_Men	15	temp_index_2	1.283	1.155	1.411	1.034	0.860	1.208
Circulatory	Age65_Men	15	temp_index_3	1.428	1.209	1.648	1.055	0.753	1.356
Circulatory	Age65_Men	15	temp_index_4	1.418	1.240	1.597	1.087	0.922	1.252
Circulatory	Age65_Men	20	temp_index_1	1.522	1.277	1.767	1.154	0.889	1.419
Circulatory	Age65_Men	20	temp_index_2	1.366	1.167	1.566	1.007	0.867	1.147
Circulatory	Age65_Men	20	temp_index_3	1.169	1.016	1.322	1.044	0.684	1.405
Circulatory	Age65_Men	20	temp_index_4	1.520	1.286	1.755	1.150	0.911	1.389
Circulatory	Age0_14_Women	3	temp_index_1	0.590	0.240	0.941	0.284	-0.046	0.614
Circulatory	Age0_14_Women	3	temp_index_2	0.652	0.300	1.003	0.755	-1.203	2.714
Circulatory	Age0_14_Women	3	temp_index_3	0.591	0.251	0.931	0.315	-0.067	0.697

Circulatory	Age0_14_Women	3	temp_index_4	0.605	0.113	1.096	0.345	-0.497	1.188
Circulatory	Age0_14_Women	5	temp_index_1	0.614	0.227	1.001	0.337	-0.099	0.773
Circulatory	Age0_14_Women	5	temp_index_2	0.670	0.291	1.048	0.447	0.119	0.775
Circulatory	Age0_14_Women	5	temp_index_3	0.469	0.032	0.907	0.345	-0.173	0.864
Circulatory	Age0_14_Women	5	temp_index_4	0.595	0.095	1.096	0.334	-0.519	1.187
Circulatory	Age0_14_Women	7	temp_index_1	0.579	0.185	0.974	0.287	-0.176	0.750
Circulatory	Age0_14_Women	7	temp_index_2	0.667	0.274	1.060	0.239	-0.110	0.589
Circulatory	Age0_14_Women	7	temp_index_3	0.603	0.213	0.993	0.283	-0.275	0.840
Circulatory	Age0_14_Women	7	temp_index_4	0.566	0.053	1.078	0.307	-0.441	1.055
Circulatory	Age0_14_Women	10	temp_index_1	0.433	-0.025	0.890	0.347	-0.512	1.206
Circulatory	Age0_14_Women	10	temp_index_2	0.647	0.248	1.046	0.315	-0.257	0.887
Circulatory	Age0_14_Women	10	temp_index_3	0.382	-0.098	0.861	0.258	-1.769	2.284
Circulatory	Age0_14_Women	10	temp_index_4	0.509	-0.026	1.044	0.350	-0.404	1.105
Circulatory	Age0_14_Women	15	temp_index_1	0.584	0.260	0.909	1.00000000000008e-10	0.000	8.76532540576602e-06
Circulatory	Age0_14_Women	15	temp_index_2	0.603	0.269	0.937	8.23542898642832e-10	0.000	4.5223063519346e-05
Circulatory	Age0_14_Women	15	temp_index_3	0.556	0.248	0.864	1.00005473572789e-10	0.000	1.21668603849895e-05
Circulatory	Age0_14_Women	15	temp_index_4	0.600	-0.166	1.365	4.64507585606766e-10	0.000	4.52227045339692e-05
Circulatory	Age0_14_Women	20	temp_index_1	0.508	0.031	0.985	6.47698428521435e-10	0.000	4.5222887689019e-05
Circulatory	Age0_14_Women	20	temp_index_2	0.387	0.090	0.683	1.0616214302767e-09	0.000	4.52233015357443e-05
Circulatory	Age0_14_Women	20	temp_index_3	0.509	-0.173	1.191	5.01626584660423e-10	0.000	4.52227416243241e-05
Circulatory	Age0_14_Women	20	temp_index_4	0.298	0.018	0.579	8.56730741416698e-10	0.000	4.52230967088167e-05
Circulatory	Age15_45_Women	3	temp_index_1	1.062	0.951	1.173	1.405	0.510	2.300
Circulatory	Age15_45_Women	3	temp_index_2	1.052	0.972	1.132	1.137	0.754	1.519
Circulatory	Age15_45_Women	3	temp_index_3	0.829	0.649	1.008	0.999	0.608	1.390
Circulatory	Age15_45_Women	3	temp_index_4	1.060	0.963	1.157	1.508	0.705	2.311
Circulatory	Age15_45_Women	5	temp_index_1	0.878	0.681	1.076	1.090	0.703	1.477
Circulatory	Age15_45_Women	5	temp_index_2	1.058	0.852	1.263	1.082	0.702	1.462
Circulatory	Age15_45_Women	5	temp_index_3	0.913	0.684	1.141	1.061	0.578	1.544
Circulatory	Age15_45_Women	5	temp_index_4	1.124	0.908	1.341	1.462	0.713	2.212
Circulatory	Age15_45_Women	7	temp_index_1	1.115	0.953	1.277	1.651	0.080	3.222
Circulatory	Age15_45_Women	7	temp_index_2	1.086	0.838	1.334	1.267	0.669	1.865
Circulatory	Age15_45_Women	7	temp_index_3	0.957	0.690	1.223	0.997	0.422	1.572
Circulatory	Age15_45_Women	7	temp_index_4	1.124	0.994	1.255	0.763	0.330	1.195
Circulatory	Age15_45_Women	10	temp_index_1	1.169	0.831	1.508	0.583	0.191	0.975
Circulatory	Age15_45_Women	10	temp_index_2	1.128	0.814	1.443	0.639	0.369	0.908
Circulatory	Age15_45_Women	10	temp_index_3	1.224	0.849	1.598	0.398	0.013	0.783
Circulatory	Age15_45_Women	10	temp_index_4	1.195	0.877	1.513	0.794	0.087	1.501
Circulatory	Age15_45_Women	15	temp_index_1	1.167	0.713	1.621	0.378	0.015	0.741
Circulatory	Age15_45_Women	15	temp_index_2	1.166	0.727	1.606	1.557	0.197	2.918

Circulatory	Age15_45_Women	15	temp_index_3	1.181	0.675	1.687	0.308	-0.023	0.639
Circulatory	Age15_45_Women	15	temp_index_4	1.185	0.756	1.614	0.732	-0.180	1.644
Circulatory	Age15_45_Women	20	temp_index_1	0.996	0.814	1.178	0.332	0.042	0.621
Circulatory	Age15_45_Women	20	temp_index_2	1.045	0.949	1.142	0.576	-0.049	1.202
Circulatory	Age15_45_Women	20	temp_index_3	1.039	0.758	1.320	0.286	-0.032	0.603
Circulatory	Age15_45_Women	20	temp_index_4	1.043	0.846	1.239	0.382	0.007	0.756
Circulatory	Age46_65_Women	3	temp_index_1	1.228	1.071	1.385	1.041	0.880	1.202
Circulatory	Age46_65_Women	3	temp_index_2	1.219	1.113	1.325	1.064	0.901	1.227
Circulatory	Age46_65_Women	3	temp_index_3	1.243	1.074	1.412	1.015	0.823	1.207
Circulatory	Age46_65_Women	3	temp_index_4	1.223	1.082	1.364	1.014	0.898	1.131
Circulatory	Age46_65_Women	5	temp_index_1	1.161	0.995	1.327	1.011	0.830	1.191
Circulatory	Age46_65_Women	5	temp_index_2	1.129	0.993	1.264	1.022	0.903	1.140
Circulatory	Age46_65_Women	5	temp_index_3	1.140	0.992	1.287	0.979	0.761	1.197
Circulatory	Age46_65_Women	5	temp_index_4	1.162	1.011	1.313	0.989	0.864	1.115
Circulatory	Age46_65_Women	7	temp_index_1	1.126	0.973	1.280	0.947	0.751	1.143
Circulatory	Age46_65_Women	7	temp_index_2	1.164	0.994	1.333	0.952	0.828	1.075
Circulatory	Age46_65_Women	7	temp_index_3	1.166	0.994	1.337	0.952	0.695	1.209
Circulatory	Age46_65_Women	7	temp_index_4	1.219	1.031	1.408	0.933	0.798	1.069
Circulatory	Age46_65_Women	10	temp_index_1	1.246	1.054	1.437	0.943	0.699	1.187
Circulatory	Age46_65_Women	10	temp_index_2	1.175	0.956	1.393	0.908	0.768	1.047
Circulatory	Age46_65_Women	10	temp_index_3	1.249	1.062	1.437	0.991	0.604	1.377
Circulatory	Age46_65_Women	10	temp_index_4	1.240	0.974	1.506	0.935	0.753	1.117
Circulatory	Age46_65_Women	15	temp_index_1	1.438	1.168	1.707	0.955	0.431	1.479
Circulatory	Age46_65_Women	15	temp_index_2	1.280	1.039	1.521	1.002	0.692	1.312
Circulatory	Age46_65_Women	15	temp_index_3	1.397	1.131	1.663	0.670	0.218	1.123
Circulatory	Age46_65_Women	15	temp_index_4	1.395	1.119	1.671	1.024	0.545	1.504
Circulatory	Age46_65_Women	20	temp_index_1	1.404	1.108	1.700	0.575	0.244	0.905
Circulatory	Age46_65_Women	20	temp_index_2	1.393	1.076	1.710	0.838	0.560	1.116
Circulatory	Age46_65_Women	20	temp_index_3	1.460	1.134	1.786	0.650	-0.171	1.471
Circulatory	Age46_65_Women	20	temp_index_4	1.259	0.976	1.542	0.721	0.285	1.157
Circulatory	Age65_Women	3	temp_index_1	1.106	1.026	1.186	1.143	1.036	1.250
Circulatory	Age65_Women	3	temp_index_2	1.091	1.050	1.132	1.190	1.076	1.304
Circulatory	Age65_Women	3	temp_index_3	1.083	1.001	1.165	1.120	0.995	1.245
Circulatory	Age65_Women	3	temp_index_4	1.104	1.034	1.175	1.133	1.044	1.221
Circulatory	Age65_Women	5	temp_index_1	1.126	1.033	1.219	1.167	1.044	1.289
Circulatory	Age65_Women	5	temp_index_2	1.129	1.074	1.184	1.237	1.100	1.374
Circulatory	Age65_Women	5	temp_index_3	1.105	1.010	1.200	1.126	0.983	1.269
Circulatory	Age65_Women	5	temp_index_4	1.129	1.045	1.214	1.142	1.041	1.243
Circulatory	Age65_Women	7	temp_index_1	1.125	1.020	1.231	1.167	1.025	1.309

Circulatory	Age65_Women	7	temp_index_2	1.128	1.060	1.196	1.195	1.045	1.345
Circulatory	Age65_Women	7	temp_index_3	1.119	1.016	1.221	1.164	0.971	1.356
Circulatory	Age65_Women	7	temp_index_4	1.125	1.028	1.222	1.148	1.025	1.271
Circulatory	Age65_Women	10	temp_index_1	1.206	1.118	1.295	1.239	0.946	1.532
Circulatory	Age65_Women	10	temp_index_2	1.157	1.070	1.243	1.195	1.018	1.372
Circulatory	Age65_Women	10	temp_index_3	1.217	1.116	1.319	1.254	0.813	1.695
Circulatory	Age65_Women	10	temp_index_4	1.196	1.113	1.280	1.282	1.021	1.543
Circulatory	Age65_Women	15	temp_index_1	1.258	1.129	1.387	1.031	0.718	1.345
Circulatory	Age65_Women	15	temp_index_2	1.173	1.058	1.288	1.137	0.915	1.358
Circulatory	Age65_Women	15	temp_index_3	1.324	1.166	1.483	0.879	0.476	1.282
Circulatory	Age65_Women	15	temp_index_4	1.214	1.090	1.339	1.132	0.842	1.422
Circulatory	Age65_Women	20	temp_index_1	1.265	1.104	1.426	0.926	0.586	1.266
Circulatory	Age65_Women	20	temp_index_2	1.186	1.048	1.323	1.075	0.822	1.328
Circulatory	Age65_Women	20	temp_index_3	1.137	0.977	1.298	0.992	0.669	1.315
Circulatory	Age65_Women	20	temp_index_4	1.216	1.064	1.368	1.059	0.727	1.390
Respiratory	allAges_allSex	3	temp_index_1	1.002	0.993	1.011	1.324	1.146	1.501
Respiratory	allAges_allSex	3	temp_index_2	1.005	0.998	1.013	1.255	1.146	1.364
Respiratory	allAges_allSex	3	temp_index_3	1.012	0.980	1.043	1.338	1.090	1.586
Respiratory	allAges_allSex	3	temp_index_4	1.004	0.997	1.012	1.326	1.157	1.495
Respiratory	allAges_allSex	5	temp_index_1	1.046	0.979	1.113	1.320	1.124	1.515
Respiratory	allAges_allSex	5	temp_index_2	1.016	0.960	1.072	1.224	1.111	1.337
Respiratory	allAges_allSex	5	temp_index_3	1.067	0.994	1.140	1.382	1.087	1.677
Respiratory	allAges_allSex	5	temp_index_4	1.039	0.993	1.086	1.305	1.131	1.480
Respiratory	allAges_allSex	7	temp_index_1	1.057	0.979	1.134	1.320	1.085	1.556
Respiratory	allAges_allSex	7	temp_index_2	1.031	0.966	1.096	1.235	1.100	1.369
Respiratory	allAges_allSex	7	temp_index_3	1.070	0.986	1.155	1.350	0.998	1.703
Respiratory	allAges_allSex	7	temp_index_4	1.046	0.976	1.115	1.299	1.096	1.501
Respiratory	allAges_allSex	10	temp_index_1	1.096	1.003	1.190	1.309	1.028	1.590
Respiratory	allAges_allSex	10	temp_index_2	1.046	0.973	1.120	1.187	1.034	1.340
Respiratory	allAges_allSex	10	temp_index_3	1.101	0.999	1.203	1.329	0.905	1.753
Respiratory	allAges_allSex	10	temp_index_4	1.092	1.006	1.177	1.294	1.047	1.540
Respiratory	allAges_allSex	15	temp_index_1	1.185	1.058	1.313	1.385	0.969	1.801
Respiratory	allAges_allSex	15	temp_index_2	1.083	0.974	1.193	1.065	0.886	1.245
Respiratory	allAges_allSex	15	temp_index_3	1.177	1.038	1.317	1.503	0.796	2.209
Respiratory	allAges_allSex	15	temp_index_4	1.183	1.065	1.301	1.330	0.986	1.673
Respiratory	allAges_allSex	20	temp_index_1	1.261	1.102	1.419	1.270	0.802	1.737
Respiratory	allAges_allSex	20	temp_index_2	1.125	0.967	1.284	0.925	0.797	1.053
Respiratory	allAges_allSex	20	temp_index_3	1.263	1.084	1.443	1.251	0.487	2.015
Respiratory	allAges_allSex	20	temp_index_4	1.251	1.108	1.395	1.250	0.865	1.635

Respiratory	allAges_Men	3	temp_index_1	1.005	0.991	1.019	1.335	1.083	1.587
Respiratory	allAges_Men	3	temp_index_2	1.010	0.999	1.021	1.235	1.093	1.377
Respiratory	allAges_Men	3	temp_index_3	1.017	0.967	1.068	1.351	0.986	1.716
Respiratory	allAges_Men	3	temp_index_4	0.986	0.951	1.022	1.310	1.079	1.541
Respiratory	allAges_Men	5	temp_index_1	0.994	0.946	1.041	1.226	0.970	1.483
Respiratory	allAges_Men	5	temp_index_2	1.021	0.943	1.099	1.167	1.036	1.298
Respiratory	allAges_Men	5	temp_index_3	1.026	0.961	1.090	1.223	0.861	1.585
Respiratory	allAges_Men	5	temp_index_4	1.034	0.955	1.113	1.229	1.000	1.457
Respiratory	allAges_Men	7	temp_index_1	1.066	0.962	1.170	1.247	0.932	1.563
Respiratory	allAges_Men	7	temp_index_2	1.033	0.947	1.119	1.193	1.016	1.371
Respiratory	allAges_Men	7	temp_index_3	1.086	0.972	1.201	1.206	0.757	1.654
Respiratory	allAges_Men	7	temp_index_4	1.070	0.976	1.164	1.242	0.964	1.519
Respiratory	allAges_Men	10	temp_index_1	1.094	0.968	1.221	1.106	0.782	1.429
Respiratory	allAges_Men	10	temp_index_2	1.009	0.963	1.055	1.073	0.891	1.255
Respiratory	allAges_Men	10	temp_index_3	1.108	0.967	1.248	1.039	0.592	1.486
Respiratory	allAges_Men	10	temp_index_4	1.104	0.987	1.220	1.099	0.808	1.389
Respiratory	allAges_Men	15	temp_index_1	1.187	1.018	1.357	1.196	0.671	1.722
Respiratory	allAges_Men	15	temp_index_2	1.068	0.923	1.212	0.970	0.738	1.203
Respiratory	allAges_Men	15	temp_index_3	1.185	0.997	1.374	1.227	0.371	2.083
Respiratory	allAges_Men	15	temp_index_4	1.210	1.050	1.370	1.153	0.721	1.584
Respiratory	allAges_Men	20	temp_index_1	1.196	1.003	1.389	1.092	0.492	1.692
Respiratory	allAges_Men	20	temp_index_2	1.048	0.884	1.213	0.984	0.691	1.278
Respiratory	allAges_Men	20	temp_index_3	1.244	1.023	1.464	0.594	0.367	0.821
Respiratory	allAges_Men	20	temp_index_4	1.247	1.068	1.425	1.075	0.576	1.573
Respiratory	allAges_Women	3	temp_index_1	1.004	0.965	1.043	1.323	1.059	1.588
Respiratory	allAges_Women	3	temp_index_2	1.004	0.995	1.012	1.312	1.124	1.501
Respiratory	allAges_Women	3	temp_index_3	1.016	0.968	1.063	1.333	0.968	1.698
Respiratory	allAges_Women	3	temp_index_4	1.005	0.998	1.012	1.339	1.081	1.597
Respiratory	allAges_Women	5	temp_index_1	1.062	0.981	1.144	1.479	1.138	1.821
Respiratory	allAges_Women	5	temp_index_2	1.030	0.965	1.094	1.361	1.144	1.579
Respiratory	allAges_Women	5	temp_index_3	1.074	0.993	1.156	1.619	1.072	2.167
Respiratory	allAges_Women	5	temp_index_4	1.053	0.997	1.110	1.445	1.147	1.743
Respiratory	allAges_Women	7	temp_index_1	1.073	0.978	1.167	1.415	1.027	1.803
Respiratory	allAges_Women	7	temp_index_2	1.047	0.970	1.124	1.314	1.077	1.552
Respiratory	allAges_Women	7	temp_index_3	1.065	0.959	1.171	1.513	0.896	2.129
Respiratory	allAges_Women	7	temp_index_4	1.047	0.973	1.122	1.393	1.066	1.719
Respiratory	allAges_Women	10	temp_index_1	1.083	0.973	1.192	1.611	1.041	2.182
Respiratory	allAges_Women	10	temp_index_2	1.058	0.966	1.149	1.388	1.074	1.703
Respiratory	allAges_Women	10	temp_index_3	1.069	0.952	1.186	1.801	0.792	2.811

Respiratory	allAges_Women	10	temp_index_4	1.044	0.951	1.138	1.533	1.082	1.983
Respiratory	allAges_Women	15	temp_index_1	1.163	0.968	1.359	1.662	0.906	2.418
Respiratory	allAges_Women	15	temp_index_2	1.070	0.943	1.198	1.245	0.885	1.605
Respiratory	allAges_Women	15	temp_index_3	1.164	0.957	1.372	1.792	0.405	3.178
Respiratory	allAges_Women	15	temp_index_4	1.149	0.980	1.317	1.626	0.982	2.269
Respiratory	allAges_Women	20	temp_index_1	1.360	1.090	1.631	1.528	0.618	2.438
Respiratory	allAges_Women	20	temp_index_2	1.267	0.982	1.553	0.937	0.741	1.134
Respiratory	allAges_Women	20	temp_index_3	1.392	1.092	1.691	0.768	0.194	1.342
Respiratory	allAges_Women	20	temp_index_4	1.285	1.053	1.517	1.562	0.767	2.357
Respiratory	Age0_14_allSex	3	temp_index_1	1.025	0.955	1.095	1.315	0.654	1.977
Respiratory	Age0_14_allSex	3	temp_index_2	1.015	0.969	1.061	1.224	0.820	1.627
Respiratory	Age0_14_allSex	3	temp_index_3	0.964	0.779	1.149	1.028	0.257	1.798
Respiratory	Age0_14_allSex	3	temp_index_4	1.040	0.967	1.112	1.233	0.667	1.798
Respiratory	Age0_14_allSex	5	temp_index_1	1.058	0.868	1.248	0.982	0.477	1.487
Respiratory	Age0_14_allSex	5	temp_index_2	0.983	0.922	1.043	1.018	0.701	1.336
Respiratory	Age0_14_allSex	5	temp_index_3	0.945	0.744	1.147	0.926	0.159	1.694
Respiratory	Age0_14_allSex	5	temp_index_4	1.011	0.972	1.051	0.958	0.474	1.441
Respiratory	Age0_14_allSex	7	temp_index_1	0.990	0.718	1.261	0.619	0.229	1.010
Respiratory	Age0_14_allSex	7	temp_index_2	1.029	0.916	1.142	1.117	0.572	1.661
Respiratory	Age0_14_allSex	7	temp_index_3	1.023	0.759	1.288	0.612	-0.159	1.383
Respiratory	Age0_14_allSex	7	temp_index_4	1.061	0.911	1.211	0.699	0.220	1.178
Respiratory	Age0_14_allSex	10	temp_index_1	1.017	0.688	1.346	1.060	0.233	1.887
Respiratory	Age0_14_allSex	10	temp_index_2	1.133	0.939	1.326	0.994	0.515	1.473
Respiratory	Age0_14_allSex	10	temp_index_3	1.106	0.742	1.469	0.744	-0.570	2.058
Respiratory	Age0_14_allSex	10	temp_index_4	1.182	0.957	1.408	0.716	-0.065	1.498
Respiratory	Age0_14_allSex	15	temp_index_1	1.370	0.929	1.811	1.508	-0.623	3.639
Respiratory	Age0_14_allSex	15	temp_index_2	1.306	0.977	1.634	1.889	0.392	3.385
Respiratory	Age0_14_allSex	15	temp_index_3	1.302	0.778	1.826	0.570	-0.415	1.556
Respiratory	Age0_14_allSex	15	temp_index_4	1.353	0.993	1.712	0.709	-0.118	1.537
Respiratory	Age0_14_allSex	20	temp_index_1	1.302	0.787	1.817	0.506	-0.493	1.506
Respiratory	Age0_14_allSex	20	temp_index_2	1.299	0.865	1.732	1.024	-0.224	2.271
Respiratory	Age0_14_allSex	20	temp_index_3	1.401	0.631	2.171	0.438	-0.561	1.437
Respiratory	Age0_14_allSex	20	temp_index_4	1.061	0.716	1.405	0.583	-0.374	1.540
Respiratory	Age15_45_allSex	3	temp_index_1	1.059	0.872	1.247	1.232	0.798	1.667
Respiratory	Age15_45_allSex	3	temp_index_2	0.997	0.928	1.066	1.091	0.822	1.360
Respiratory	Age15_45_allSex	3	temp_index_3	1.067	0.878	1.256	1.384	0.553	2.214
Respiratory	Age15_45_allSex	3	temp_index_4	1.093	0.911	1.274	1.328	0.758	1.897
Respiratory	Age15_45_allSex	5	temp_index_1	1.141	0.871	1.412	1.155	0.731	1.580
Respiratory	Age15_45_allSex	5	temp_index_2	1.155	0.911	1.399	1.085	0.801	1.370

Respiratory	Age15_45_allSex	5	temp_index_3	1.143	0.864	1.421	1.255	0.637	1.872
Respiratory	Age15_45_allSex	5	temp_index_4	1.266	0.969	1.564	1.153	0.685	1.621
Respiratory	Age15_45_allSex	7	temp_index_1	1.462	1.026	1.898	1.206	0.544	1.867
Respiratory	Age15_45_allSex	7	temp_index_2	1.230	0.923	1.538	1.117	0.740	1.493
Respiratory	Age15_45_allSex	7	temp_index_3	1.193	0.840	1.546	1.210	0.532	1.888
Respiratory	Age15_45_allSex	7	temp_index_4	1.394	1.035	1.754	1.113	0.422	1.804
Respiratory	Age15_45_allSex	10	temp_index_1	1.495	0.953	2.038	1.073	0.312	1.834
Respiratory	Age15_45_allSex	10	temp_index_2	1.228	0.893	1.563	1.049	0.642	1.456
Respiratory	Age15_45_allSex	10	temp_index_3	0.990	0.547	1.433	0.755	0.270	1.240
Respiratory	Age15_45_allSex	10	temp_index_4	1.409	0.975	1.842	1.054	0.200	1.908
Respiratory	Age15_45_allSex	15	temp_index_1	1.683	0.911	2.455	0.572	0.114	1.031
Respiratory	Age15_45_allSex	15	temp_index_2	1.234	0.859	1.610	0.897	0.493	1.302
Respiratory	Age15_45_allSex	15	temp_index_3	1.687	0.685	2.688	0.499	0.076	0.923
Respiratory	Age15_45_allSex	15	temp_index_4	1.385	0.861	1.909	0.554	0.182	0.926
Respiratory	Age15_45_allSex	20	temp_index_1	1.776	0.596	2.956	0.748	0.060	1.435
Respiratory	Age15_45_allSex	20	temp_index_2	1.305	0.726	1.884	0.754	0.413	1.095
Respiratory	Age15_45_allSex	20	temp_index_3	1.598	0.123	3.073	0.380	-0.182	0.941
Respiratory	Age15_45_allSex	20	temp_index_4	1.564	0.764	2.365	0.768	0.222	1.314
Respiratory	Age46_65_allSex	3	temp_index_1	1.114	0.967	1.261	1.205	0.774	1.637
Respiratory	Age46_65_allSex	3	temp_index_2	1.021	0.962	1.079	1.059	0.837	1.280
Respiratory	Age46_65_allSex	3	temp_index_3	1.130	0.967	1.292	1.296	0.658	1.934
Respiratory	Age46_65_allSex	3	temp_index_4	1.077	0.957	1.198	1.111	0.760	1.462
Respiratory	Age46_65_allSex	5	temp_index_1	1.170	0.982	1.358	1.247	0.725	1.769
Respiratory	Age46_65_allSex	5	temp_index_2	1.070	0.939	1.201	1.039	0.800	1.277
Respiratory	Age46_65_allSex	5	temp_index_3	1.184	0.951	1.418	1.452	0.636	2.267
Respiratory	Age46_65_allSex	5	temp_index_4	1.127	0.971	1.283	1.161	0.745	1.576
Respiratory	Age46_65_allSex	7	temp_index_1	1.136	0.896	1.375	1.184	0.637	1.731
Respiratory	Age46_65_allSex	7	temp_index_2	1.071	0.924	1.217	1.013	0.781	1.244
Respiratory	Age46_65_allSex	7	temp_index_3	1.156	0.910	1.401	0.733	0.351	1.115
Respiratory	Age46_65_allSex	7	temp_index_4	1.154	0.964	1.344	1.164	0.676	1.652
Respiratory	Age46_65_allSex	10	temp_index_1	1.169	0.890	1.447	1.255	0.525	1.984
Respiratory	Age46_65_allSex	10	temp_index_2	1.113	0.790	1.437	0.948	0.755	1.141
Respiratory	Age46_65_allSex	10	temp_index_3	1.265	0.875	1.654	0.983	0.582	1.384
Respiratory	Age46_65_allSex	10	temp_index_4	1.208	0.973	1.443	1.260	0.571	1.950
Respiratory	Age46_65_allSex	15	temp_index_1	1.156	0.809	1.503	1.611	0.120	3.102
Respiratory	Age46_65_allSex	15	temp_index_2	0.990	0.651	1.329	0.917	0.631	1.203
Respiratory	Age46_65_allSex	15	temp_index_3	1.153	0.787	1.519	0.488	-0.129	1.106
Respiratory	Age46_65_allSex	15	temp_index_4	1.211	0.916	1.506	0.612	0.342	0.883
Respiratory	Age46_65_allSex	20	temp_index_1	1.154	0.739	1.570	0.623	0.120	1.126

Respiratory	Age46_65_allSex	20	temp_index_2	0.704	0.591	0.817	1.019	0.580	1.458
Respiratory	Age46_65_allSex	20	temp_index_3	1.232	0.721	1.743	0.358	-0.032	0.747
Respiratory	Age46_65_allSex	20	temp_index_4	1.206	0.789	1.623	0.665	0.240	1.089
Respiratory	Age65_allSex	3	temp_index_1	1.002	0.993	1.012	1.394	1.147	1.641
Respiratory	Age65_allSex	3	temp_index_2	1.006	0.998	1.014	1.374	1.206	1.542
Respiratory	Age65_allSex	3	temp_index_3	1.005	0.995	1.015	1.360	1.017	1.703
Respiratory	Age65_allSex	3	temp_index_4	1.008	1.001	1.015	1.382	1.158	1.605
Respiratory	Age65_allSex	5	temp_index_1	0.999	0.955	1.043	1.391	1.108	1.673
Respiratory	Age65_allSex	5	temp_index_2	0.994	0.964	1.024	1.366	1.181	1.551
Respiratory	Age65_allSex	5	temp_index_3	1.027	0.967	1.087	1.412	1.011	1.813
Respiratory	Age65_allSex	5	temp_index_4	1.014	0.959	1.068	1.368	1.140	1.595
Respiratory	Age65_allSex	7	temp_index_1	1.044	0.962	1.126	1.366	1.056	1.676
Respiratory	Age65_allSex	7	temp_index_2	1.011	0.944	1.078	1.362	1.157	1.566
Respiratory	Age65_allSex	7	temp_index_3	1.059	0.959	1.158	1.357	0.904	1.810
Respiratory	Age65_allSex	7	temp_index_4	1.026	0.952	1.100	1.362	1.100	1.624
Respiratory	Age65_allSex	10	temp_index_1	1.037	0.970	1.104	1.390	0.975	1.806
Respiratory	Age65_allSex	10	temp_index_2	1.015	0.971	1.060	1.376	1.109	1.643
Respiratory	Age65_allSex	10	temp_index_3	1.064	0.937	1.192	1.333	0.774	1.892
Respiratory	Age65_allSex	10	temp_index_4	1.057	0.962	1.152	1.335	1.018	1.651
Respiratory	Age65_allSex	15	temp_index_1	1.166	1.008	1.323	1.394	0.857	1.931
Respiratory	Age65_allSex	15	temp_index_2	1.059	0.941	1.177	1.185	0.907	1.462
Respiratory	Age65_allSex	15	temp_index_3	1.185	1.007	1.363	1.454	0.544	2.363
Respiratory	Age65_allSex	15	temp_index_4	1.183	1.046	1.319	1.361	0.912	1.809
Respiratory	Age65_allSex	20	temp_index_1	1.315	1.103	1.527	1.308	0.684	1.932
Respiratory	Age65_allSex	20	temp_index_2	1.103	0.959	1.247	1.104	0.782	1.427
Respiratory	Age65_allSex	20	temp_index_3	1.334	1.085	1.583	1.257	0.221	2.293
Respiratory	Age65_allSex	20	temp_index_4	1.310	1.131	1.488	1.283	0.758	1.808
Respiratory	Age0_14_Men	3	temp_index_1	1.049	0.782	1.316	0.928	0.205	1.651
Respiratory	Age0_14_Men	3	temp_index_2	0.862	0.625	1.099	0.963	0.595	1.332
Respiratory	Age0_14_Men	3	temp_index_3	1.029	0.748	1.310	0.436	0.126	0.745
Respiratory	Age0_14_Men	3	temp_index_4	1.010	0.754	1.267	0.791	0.315	1.266
Respiratory	Age0_14_Men	5	temp_index_1	0.913	0.654	1.172	0.869	0.094	1.643
Respiratory	Age0_14_Men	5	temp_index_2	0.998	0.915	1.082	0.992	0.561	1.423
Respiratory	Age0_14_Men	5	temp_index_3	0.917	0.655	1.178	0.419	0.068	0.770
Respiratory	Age0_14_Men	5	temp_index_4	1.039	0.925	1.152	0.905	0.182	1.628
Respiratory	Age0_14_Men	7	temp_index_1	0.987	0.646	1.327	0.730	-0.179	1.640
Respiratory	Age0_14_Men	7	temp_index_2	0.859	0.557	1.162	0.801	0.444	1.159
Respiratory	Age0_14_Men	7	temp_index_3	1.028	0.669	1.387	0.303	0.059	0.547
Respiratory	Age0_14_Men	7	temp_index_4	0.886	0.570	1.202	0.766	-0.041	1.574

Respiratory	Age0_14_Men	10	temp_index_1	0.969	0.545	1.393	1.043	-0.753	2.840
Respiratory	Age0_14_Men	10	temp_index_2	0.865	0.506	1.224	0.961	0.357	1.564
Respiratory	Age0_14_Men	10	temp_index_3	1.030	0.568	1.492	0.302	-0.018	0.622
Respiratory	Age0_14_Men	10	temp_index_4	0.897	0.480	1.314	1.059	-0.696	2.815
Respiratory	Age0_14_Men	15	temp_index_1	1.029	0.495	1.564	0.787	-0.615	2.189
Respiratory	Age0_14_Men	15	temp_index_2	1.075	0.890	1.260	1.133	0.198	2.068
Respiratory	Age0_14_Men	15	temp_index_3	1.154	0.517	1.792	0.711	-1.143	2.565
Respiratory	Age0_14_Men	15	temp_index_4	0.406	0.260	0.552	0.876	-0.515	2.267
Respiratory	Age0_14_Men	20	temp_index_1	1.007	0.331	1.683	0.525	-0.920	1.970
Respiratory	Age0_14_Men	20	temp_index_2	1.133	0.675	1.592	0.621	0.037	1.206
Respiratory	Age0_14_Men	20	temp_index_3	1.258	0.336	2.179	0.333	-1.255	1.922
Respiratory	Age0_14_Men	20	temp_index_4	0.413	0.271	0.556	0.819	-1.188	2.827
Respiratory	Age15_45_Men	3	temp_index_1	1.242	0.884	1.601	1.228	0.650	1.807
Respiratory	Age15_45_Men	3	temp_index_2	1.146	0.925	1.368	1.110	0.623	1.598
Respiratory	Age15_45_Men	3	temp_index_3	1.222	0.887	1.556	1.359	0.191	2.528
Respiratory	Age15_45_Men	3	temp_index_4	1.215	0.854	1.576	1.206	0.588	1.825
Respiratory	Age15_45_Men	5	temp_index_1	1.311	0.852	1.769	1.236	0.614	1.859
Respiratory	Age15_45_Men	5	temp_index_2	1.104	0.793	1.415	1.062	0.718	1.405
Respiratory	Age15_45_Men	5	temp_index_3	1.265	0.813	1.718	1.314	0.341	2.286
Respiratory	Age15_45_Men	5	temp_index_4	1.228	0.839	1.616	1.068	0.655	1.482
Respiratory	Age15_45_Men	7	temp_index_1	1.386	0.762	2.009	1.110	0.455	1.765
Respiratory	Age15_45_Men	7	temp_index_2	0.892	0.529	1.254	0.900	0.547	1.253
Respiratory	Age15_45_Men	7	temp_index_3	1.363	0.623	2.103	1.011	0.148	1.875
Respiratory	Age15_45_Men	7	temp_index_4	1.793	1.028	2.557	0.988	0.448	1.528
Respiratory	Age15_45_Men	10	temp_index_1	1.581	0.690	2.472	0.996	0.279	1.713
Respiratory	Age15_45_Men	10	temp_index_2	0.899	0.517	1.281	0.886	0.441	1.330
Respiratory	Age15_45_Men	10	temp_index_3	1.537	0.408	2.665	0.877	-0.096	1.850
Respiratory	Age15_45_Men	10	temp_index_4	1.681	0.888	2.474	0.963	0.028	1.898
Respiratory	Age15_45_Men	15	temp_index_1	0.609	0.180	1.039	0.706	0.095	1.317
Respiratory	Age15_45_Men	15	temp_index_2	1.493	0.717	2.269	0.803	0.226	1.381
Respiratory	Age15_45_Men	15	temp_index_3	1.662	-0.185	3.510	0.688	-0.282	1.658
Respiratory	Age15_45_Men	15	temp_index_4	1.366	0.261	2.472	0.441	0.047	0.835
Respiratory	Age15_45_Men	20	temp_index_1	0.723	0.049	1.396	0.451	-0.378	1.280
Respiratory	Age15_45_Men	20	temp_index_2	1.561	0.276	2.846	0.599	0.205	0.994
Respiratory	Age15_45_Men	20	temp_index_3	0.646	0.071	1.221	0.435	-0.636	1.505
Respiratory	Age15_45_Men	20	temp_index_4	1.812	0.022	3.603	0.376	-0.187	0.939
Respiratory	Age46_65_Men	3	temp_index_1	0.977	0.892	1.063	0.683	0.485	0.881
Respiratory	Age46_65_Men	3	temp_index_2	0.981	0.918	1.044	1.359	0.908	1.810
Respiratory	Age46_65_Men	3	temp_index_3	1.049	0.906	1.192	0.639	0.423	0.854

Respiratory	Age46_65_Men	3	temp_index_4	0.978	0.904	1.053	0.725	0.534	0.917
Respiratory	Age46_65_Men	5	temp_index_1	0.955	0.869	1.040	0.637	0.442	0.831
Respiratory	Age46_65_Men	5	temp_index_2	0.953	0.891	1.014	1.123	0.721	1.525
Respiratory	Age46_65_Men	5	temp_index_3	1.061	0.878	1.243	0.601	0.385	0.817
Respiratory	Age46_65_Men	5	temp_index_4	0.971	0.890	1.052	0.695	0.503	0.886
Respiratory	Age46_65_Men	7	temp_index_1	0.926	0.844	1.007	0.659	0.418	0.901
Respiratory	Age46_65_Men	7	temp_index_2	0.942	0.877	1.007	1.018	0.631	1.405
Respiratory	Age46_65_Men	7	temp_index_3	0.987	0.697	1.276	0.624	0.329	0.919
Respiratory	Age46_65_Men	7	temp_index_4	0.951	0.868	1.034	0.764	0.498	1.029
Respiratory	Age46_65_Men	10	temp_index_1	0.940	0.589	1.290	0.941	0.392	1.490
Respiratory	Age46_65_Men	10	temp_index_2	0.731	0.551	0.911	1.024	0.669	1.378
Respiratory	Age46_65_Men	10	temp_index_3	0.753	0.515	0.990	0.933	0.408	1.457
Respiratory	Age46_65_Men	10	temp_index_4	0.732	0.561	0.903	0.950	0.697	1.203
Respiratory	Age46_65_Men	15	temp_index_1	0.823	0.526	1.120	0.773	0.333	1.214
Respiratory	Age46_65_Men	15	temp_index_2	0.930	0.816	1.044	0.839	0.565	1.113
Respiratory	Age46_65_Men	15	temp_index_3	1.010	0.456	1.563	0.868	0.079	1.657
Respiratory	Age46_65_Men	15	temp_index_4	1.039	0.774	1.304	0.772	0.516	1.028
Respiratory	Age46_65_Men	20	temp_index_1	1.027	0.745	1.309	0.798	0.165	1.430
Respiratory	Age46_65_Men	20	temp_index_2	0.803	0.571	1.034	1.092	0.461	1.723
Respiratory	Age46_65_Men	20	temp_index_3	1.225	0.340	2.110	0.230	0.050	0.410
Respiratory	Age46_65_Men	20	temp_index_4	1.179	0.811	1.547	0.778	0.402	1.155
Respiratory	Age65_Men	3	temp_index_1	1.000	0.983	1.017	1.348	1.033	1.663
Respiratory	Age65_Men	3	temp_index_2	1.008	0.995	1.022	1.330	1.122	1.539
Respiratory	Age65_Men	3	temp_index_3	1.005	0.988	1.021	1.353	0.857	1.848
Respiratory	Age65_Men	3	temp_index_4	0.977	0.933	1.022	1.316	1.033	1.599
Respiratory	Age65_Men	5	temp_index_1	0.986	0.925	1.046	1.288	0.943	1.633
Respiratory	Age65_Men	5	temp_index_2	1.021	0.951	1.091	1.330	1.095	1.564
Respiratory	Age65_Men	5	temp_index_3	1.037	0.912	1.162	1.254	0.759	1.749
Respiratory	Age65_Men	5	temp_index_4	1.039	0.962	1.116	1.297	0.989	1.604
Respiratory	Age65_Men	7	temp_index_1	1.052	0.919	1.184	1.405	0.937	1.872
Respiratory	Age65_Men	7	temp_index_2	1.032	0.951	1.112	1.444	1.121	1.767
Respiratory	Age65_Men	7	temp_index_3	1.056	0.909	1.202	1.371	0.656	2.087
Respiratory	Age65_Men	7	temp_index_4	1.065	0.962	1.168	1.400	0.992	1.807
Respiratory	Age65_Men	10	temp_index_1	1.041	0.943	1.139	1.308	0.697	1.919
Respiratory	Age65_Men	10	temp_index_2	1.000	0.955	1.044	1.395	0.986	1.805
Respiratory	Age65_Men	10	temp_index_3	1.083	0.905	1.262	0.778	0.539	1.017
Respiratory	Age65_Men	10	temp_index_4	1.101	0.954	1.247	1.227	0.777	1.676
Respiratory	Age65_Men	15	temp_index_1	1.183	0.967	1.399	1.409	0.514	2.304
Respiratory	Age65_Men	15	temp_index_2	1.067	0.901	1.233	1.386	0.855	1.917

Respiratory	Age65_Men	15	temp_index_3	1.173	0.939	1.406	0.706	0.368	1.043
Respiratory	Age65_Men	15	temp_index_4	1.230	1.035	1.424	1.348	0.642	2.054
Respiratory	Age65_Men	20	temp_index_1	1.218	0.957	1.479	0.825	0.442	1.208
Respiratory	Age65_Men	20	temp_index_2	1.040	0.827	1.254	1.465	0.748	2.183
Respiratory	Age65_Men	20	temp_index_3	1.211	0.932	1.490	0.752	0.324	1.179
Respiratory	Age65_Men	20	temp_index_4	1.269	1.025	1.513	0.766	0.473	1.059
Respiratory	Age0_14_Women	3	temp_index_1	1.007	0.920	1.094	1.775	0.250	3.300
Respiratory	Age0_14_Women	3	temp_index_2	1.017	0.963	1.072	1.864	0.745	2.983
Respiratory	Age0_14_Women	3	temp_index_3	0.943	0.798	1.087	1.297	-0.083	2.677
Respiratory	Age0_14_Women	3	temp_index_4	1.024	0.939	1.108	1.495	0.403	2.586
Respiratory	Age0_14_Women	5	temp_index_1	1.119	0.789	1.450	1.173	0.177	2.168
Respiratory	Age0_14_Women	5	temp_index_2	1.001	0.984	1.019	0.967	0.268	1.666
Respiratory	Age0_14_Women	5	temp_index_3	1.037	0.703	1.371	0.983	-0.086	2.052
Respiratory	Age0_14_Women	5	temp_index_4	1.043	0.862	1.225	1.090	0.184	1.995
Respiratory	Age0_14_Women	7	temp_index_1	1.137	0.737	1.536	0.553	-0.114	1.221
Respiratory	Age0_14_Women	7	temp_index_2	1.023	0.726	1.321	0.614	0.083	1.146
Respiratory	Age0_14_Women	7	temp_index_3	1.052	0.661	1.444	0.447	-0.123	1.016
Respiratory	Age0_14_Women	7	temp_index_4	1.077	0.806	1.347	0.582	-0.271	1.434
Respiratory	Age0_14_Women	10	temp_index_1	1.296	0.794	1.798	0.944	-0.774	2.663
Respiratory	Age0_14_Women	10	temp_index_2	1.231	0.884	1.578	1.012	-0.139	2.163
Respiratory	Age0_14_Women	10	temp_index_3	1.284	0.727	1.841	0.480	-0.071	1.030
Respiratory	Age0_14_Women	10	temp_index_4	1.320	0.865	1.776	0.732	-0.698	2.162
Respiratory	Age0_14_Women	15	temp_index_1	0.973	0.422	1.523	0.167	-0.007	0.342
Respiratory	Age0_14_Women	15	temp_index_2	0.832	0.667	0.997	0.929	-0.749	2.607
Respiratory	Age0_14_Women	15	temp_index_3	0.977	0.212	1.743	0.149	-0.029	0.327
Respiratory	Age0_14_Women	15	temp_index_4	0.732	0.609	0.854	0.750	-0.976	2.476
Respiratory	Age0_14_Women	20	temp_index_1	1.026	0.226	1.826	0.213	-0.046	0.472
Respiratory	Age0_14_Women	20	temp_index_2	0.794	0.608	0.980	0.388	-0.541	1.317
Respiratory	Age0_14_Women	20	temp_index_3	1.242	0.167	2.318	0.000	-0.010	0.011
Respiratory	Age0_14_Women	20	temp_index_4	0.684	0.569	0.799	0.418	-1.161	1.996
Respiratory	Age15_45_Women	3	temp_index_1	0.845	0.522	1.168	1.102	-0.124	2.328
Respiratory	Age15_45_Women	3	temp_index_2	1.025	0.967	1.083	1.605	0.521	2.689
Respiratory	Age15_45_Women	3	temp_index_3	0.856	0.566	1.145	0.842	0.200	1.484
Respiratory	Age15_45_Women	3	temp_index_4	1.011	0.946	1.076	1.292	-0.190	2.775
Respiratory	Age15_45_Women	5	temp_index_1	1.172	0.785	1.559	0.841	0.098	1.584
Respiratory	Age15_45_Women	5	temp_index_2	1.082	0.876	1.288	1.290	0.418	2.162
Respiratory	Age15_45_Women	5	temp_index_3	1.040	0.666	1.414	1.196	-0.165	2.556
Respiratory	Age15_45_Women	5	temp_index_4	1.101	0.768	1.433	0.938	0.179	1.696
Respiratory	Age15_45_Women	7	temp_index_1	1.128	0.757	1.499	1.107	-0.440	2.655

Respiratory	Age15_45_Women	7	temp_index_2	1.016	0.916	1.117	1.581	-0.207	3.368
Respiratory	Age15_45_Women	7	temp_index_3	1.023	0.664	1.382	0.429	-0.262	1.120
Respiratory	Age15_45_Women	7	temp_index_4	1.064	0.767	1.360	1.498	-0.672	3.668
Respiratory	Age15_45_Women	10	temp_index_1	1.232	0.562	1.902	0.974	-0.542	2.491
Respiratory	Age15_45_Women	10	temp_index_2	1.100	0.774	1.426	1.143	-0.456	2.741
Respiratory	Age15_45_Women	10	temp_index_3	1.034	0.642	1.427	0.895	-0.518	2.309
Respiratory	Age15_45_Women	10	temp_index_4	1.249	0.694	1.805	1.089	-1.013	3.191
Respiratory	Age15_45_Women	15	temp_index_1	1.314	0.343	2.286	-0.002	-0.233	0.230
Respiratory	Age15_45_Women	15	temp_index_2	1.215	0.690	1.741	0.603	-0.119	1.324
Respiratory	Age15_45_Women	15	temp_index_3	1.428	0.029	2.828	0.546	0.047	1.045
Respiratory	Age15_45_Women	15	temp_index_4	1.218	0.484	1.952	0.436	-0.152	1.024
Respiratory	Age15_45_Women	20	temp_index_1	0.798	-0.198	1.794	0.481	0.072	0.889
Respiratory	Age15_45_Women	20	temp_index_2	1.098	0.351	1.845	0.741	-0.608	2.089
Respiratory	Age15_45_Women	20	temp_index_3	0.430	0.024	0.836	0.458	0.002	0.914
Respiratory	Age15_45_Women	20	temp_index_4	0.840	0.078	1.602	0.448	0.144	0.752
Respiratory	Age46_65_Women	3	temp_index_1	1.023	0.745	1.301	1.121	0.613	1.629
Respiratory	Age46_65_Women	3	temp_index_2	1.100	0.769	1.431	0.966	0.755	1.176
Respiratory	Age46_65_Women	3	temp_index_3	0.983	0.705	1.261	1.363	0.286	2.439
Respiratory	Age46_65_Women	3	temp_index_4	0.972	0.755	1.188	1.139	0.586	1.692
Respiratory	Age46_65_Women	5	temp_index_1	1.130	0.783	1.478	1.385	0.613	2.158
Respiratory	Age46_65_Women	5	temp_index_2	1.411	1.041	1.782	1.029	0.752	1.306
Respiratory	Age46_65_Women	5	temp_index_3	1.096	0.725	1.467	1.503	-0.174	3.179
Respiratory	Age46_65_Women	5	temp_index_4	1.089	0.822	1.357	1.308	0.446	2.171
Respiratory	Age46_65_Women	7	temp_index_1	1.170	0.781	1.559	1.360	0.455	2.265
Respiratory	Age46_65_Women	7	temp_index_2	1.366	0.978	1.755	1.001	0.696	1.306
Respiratory	Age46_65_Women	7	temp_index_3	1.132	0.735	1.528	1.012	0.337	1.687
Respiratory	Age46_65_Women	7	temp_index_4	1.466	1.072	1.860	0.960	0.075	1.845
Respiratory	Age46_65_Women	10	temp_index_1	1.200	0.708	1.692	1.306	0.370	2.243
Respiratory	Age46_65_Women	10	temp_index_2	1.751	1.051	2.451	1.037	0.633	1.440
Respiratory	Age46_65_Women	10	temp_index_3	1.186	0.664	1.707	0.262	0.161	0.363
Respiratory	Age46_65_Women	10	temp_index_4	1.072	0.689	1.454	1.159	0.471	1.847
Respiratory	Age46_65_Women	15	temp_index_1	1.304	0.608	2.000	0.229	0.134	0.324
Respiratory	Age46_65_Women	15	temp_index_2	1.229	0.533	1.925	1.192	0.221	2.163
Respiratory	Age46_65_Women	15	temp_index_3	1.300	0.600	2.001	0.225	0.120	0.330
Respiratory	Age46_65_Women	15	temp_index_4	1.312	0.626	1.998	0.852	0.094	1.610
Respiratory	Age46_65_Women	20	temp_index_1	1.222	0.396	2.049	0.237	0.123	0.351
Respiratory	Age46_65_Women	20	temp_index_2	1.540	0.566	2.514	1.359	-0.483	3.202
Respiratory	Age46_65_Women	20	temp_index_3	1.088	0.755	1.420	0.240	0.112	0.368
Respiratory	Age46_65_Women	20	temp_index_4	1.481	0.420	2.542	0.864	-0.478	2.207

Respiratory	Age65_Women	3	temp_index_1	1.005	0.992	1.018	1.430	1.037	1.823
Respiratory	Age65_Women	3	temp_index_2	1.005	0.995	1.015	1.432	1.155	1.708
Respiratory	Age65_Women	3	temp_index_3	1.007	0.949	1.065	1.339	0.843	1.835
Respiratory	Age65_Women	3	temp_index_4	1.006	0.999	1.012	1.467	1.096	1.838
Respiratory	Age65_Women	5	temp_index_1	1.008	0.942	1.074	1.484	1.011	1.957
Respiratory	Age65_Women	5	temp_index_2	0.995	0.952	1.039	1.427	1.109	1.744
Respiratory	Age65_Women	5	temp_index_3	1.038	0.963	1.114	0.922	0.657	1.186
Respiratory	Age65_Women	5	temp_index_4	1.015	0.953	1.076	1.478	1.097	1.859
Respiratory	Age65_Women	7	temp_index_1	1.046	0.958	1.134	1.337	0.852	1.822
Respiratory	Age65_Women	7	temp_index_2	1.029	0.971	1.087	1.333	1.023	1.643
Respiratory	Age65_Women	7	temp_index_3	1.086	0.963	1.208	0.946	0.678	1.214
Respiratory	Age65_Women	7	temp_index_4	1.014	0.944	1.083	1.334	0.952	1.716
Respiratory	Age65_Women	10	temp_index_1	1.039	0.948	1.130	1.440	0.849	2.031
Respiratory	Age65_Women	10	temp_index_2	1.018	0.958	1.077	1.396	0.990	1.802
Respiratory	Age65_Women	10	temp_index_3	1.040	0.889	1.191	1.391	0.531	2.252
Respiratory	Age65_Women	10	temp_index_4	0.984	0.880	1.088	1.428	0.937	1.919
Respiratory	Age65_Women	15	temp_index_1	1.154	0.851	1.457	1.242	0.856	1.628
Respiratory	Age65_Women	15	temp_index_2	1.032	0.791	1.273	1.132	0.880	1.384
Respiratory	Age65_Women	15	temp_index_3	0.926	0.700	1.152	1.203	0.717	1.689
Respiratory	Age65_Women	15	temp_index_4	1.137	0.903	1.372	1.383	0.782	1.985
Respiratory	Age65_Women	20	temp_index_1	1.499	1.056	1.942	1.076	0.599	1.553
Respiratory	Age65_Women	20	temp_index_2	1.260	0.920	1.599	0.935	0.701	1.168
Respiratory	Age65_Women	20	temp_index_3	1.291	0.898	1.685	1.093	0.334	1.851
Respiratory	Age65_Women	20	temp_index_4	1.422	1.011	1.833	1.153	0.593	1.712
All_cause	allAges_allSex	3	temp_index_1	1.068	1.054	1.083	1.172	1.127	1.216
All_cause	allAges_allSex	3	temp_index_2	1.044	1.032	1.056	1.146	1.116	1.175
All_cause	allAges_allSex	3	temp_index_3	1.056	1.040	1.073	1.152	1.093	1.211
All_cause	allAges_allSex	3	temp_index_4	1.066	1.053	1.079	1.176	1.135	1.217
All_cause	allAges_allSex	5	temp_index_1	1.092	1.074	1.110	1.190	1.138	1.241
All_cause	allAges_allSex	5	temp_index_2	1.062	1.047	1.077	1.141	1.108	1.174
All_cause	allAges_allSex	5	temp_index_3	1.082	1.063	1.102	1.195	1.123	1.267
All_cause	allAges_allSex	5	temp_index_4	1.087	1.071	1.103	1.187	1.141	1.233
All_cause	allAges_allSex	7	temp_index_1	1.108	1.087	1.129	1.161	1.104	1.218
All_cause	allAges_allSex	7	temp_index_2	1.074	1.055	1.092	1.112	1.076	1.149
All_cause	allAges_allSex	7	temp_index_3	1.097	1.074	1.120	1.179	1.096	1.262
All_cause	allAges_allSex	7	temp_index_4	1.093	1.073	1.114	1.146	1.096	1.197
All_cause	allAges_allSex	10	temp_index_1	1.134	1.108	1.160	1.150	1.083	1.218
All_cause	allAges_allSex	10	temp_index_2	1.095	1.071	1.118	1.088	1.046	1.130
All_cause	allAges_allSex	10	temp_index_3	1.121	1.092	1.150	1.186	1.087	1.285

All_cause	allAges_allSex	10	temp_index_4	1.132	1.107	1.158	1.148	1.087	1.209
All_cause	allAges_allSex	15	temp_index_1	1.176	1.138	1.213	1.132	1.048	1.215
All_cause	allAges_allSex	15	temp_index_2	1.124	1.090	1.158	1.065	1.015	1.114
All_cause	allAges_allSex	15	temp_index_3	1.175	1.137	1.214	1.168	1.040	1.296
All_cause	allAges_allSex	15	temp_index_4	1.177	1.143	1.211	1.125	1.050	1.200
All_cause	allAges_allSex	20	temp_index_1	1.187	1.143	1.231	1.115	1.015	1.216
All_cause	allAges_allSex	20	temp_index_2	1.121	1.081	1.162	1.040	0.984	1.096
All_cause	allAges_allSex	20	temp_index_3	1.172	1.125	1.219	1.129	0.976	1.283
All_cause	allAges_allSex	20	temp_index_4	1.185	1.144	1.226	1.108	1.018	1.197
All_cause	allAges_Men	3	temp_index_1	1.071	1.052	1.090	1.130	1.074	1.185
All_cause	allAges_Men	3	temp_index_2	1.044	1.028	1.059	1.103	1.067	1.140
All_cause	allAges_Men	3	temp_index_3	1.070	1.050	1.089	1.113	1.038	1.188
All_cause	allAges_Men	3	temp_index_4	1.068	1.051	1.085	1.134	1.083	1.185
All_cause	allAges_Men	5	temp_index_1	1.095	1.072	1.118	1.155	1.090	1.220
All_cause	allAges_Men	5	temp_index_2	1.062	1.042	1.082	1.097	1.056	1.137
All_cause	allAges_Men	5	temp_index_3	1.089	1.064	1.115	1.152	1.063	1.241
All_cause	allAges_Men	5	temp_index_4	1.076	1.055	1.098	1.140	1.083	1.197
All_cause	allAges_Men	7	temp_index_1	1.116	1.089	1.144	1.133	1.061	1.206
All_cause	allAges_Men	7	temp_index_2	1.073	1.049	1.096	1.068	1.023	1.114
All_cause	allAges_Men	7	temp_index_3	1.110	1.075	1.144	1.144	1.044	1.245
All_cause	allAges_Men	7	temp_index_4	1.102	1.076	1.129	1.112	1.048	1.176
All_cause	allAges_Men	10	temp_index_1	1.148	1.112	1.184	1.093	1.014	1.172
All_cause	allAges_Men	10	temp_index_2	1.099	1.068	1.131	1.032	0.981	1.082
All_cause	allAges_Men	10	temp_index_3	1.136	1.096	1.175	1.116	1.000	1.233
All_cause	allAges_Men	10	temp_index_4	1.135	1.102	1.168	1.077	1.005	1.149
All_cause	allAges_Men	15	temp_index_1	1.192	1.141	1.242	1.116	1.012	1.220
All_cause	allAges_Men	15	temp_index_2	1.140	1.094	1.185	1.020	0.961	1.080
All_cause	allAges_Men	15	temp_index_3	1.178	1.127	1.229	1.182	1.012	1.351
All_cause	allAges_Men	15	temp_index_4	1.188	1.142	1.235	1.100	1.008	1.193
All_cause	allAges_Men	20	temp_index_1	1.205	1.145	1.265	1.110	0.982	1.238
All_cause	allAges_Men	20	temp_index_2	1.132	1.077	1.187	1.014	0.949	1.079
All_cause	allAges_Men	20	temp_index_3	1.185	1.122	1.248	1.140	0.937	1.342
All_cause	allAges_Men	20	temp_index_4	1.204	1.147	1.261	1.095	0.981	1.208
All_cause	allAges_Women	3	temp_index_1	1.065	1.043	1.088	1.234	1.161	1.308
All_cause	allAges_Women	3	temp_index_2	1.044	1.025	1.064	1.211	1.162	1.260
All_cause	allAges_Women	3	temp_index_3	1.045	1.020	1.071	1.222	1.121	1.322
All_cause	allAges_Women	3	temp_index_4	1.063	1.042	1.084	1.238	1.171	1.306
All_cause	allAges_Women	5	temp_index_1	1.091	1.063	1.118	1.241	1.157	1.325
All_cause	allAges_Women	5	temp_index_2	1.063	1.039	1.087	1.210	1.155	1.266

All_cause	allAges_Women	5	temp_index_3	1.072	1.043	1.102	1.267	1.143	1.390
All_cause	allAges_Women	5	temp_index_4	1.089	1.064	1.115	1.241	1.166	1.317
All_cause	allAges_Women	7	temp_index_1	1.099	1.067	1.131	1.203	1.109	1.296
All_cause	allAges_Women	7	temp_index_2	1.073	1.043	1.103	1.180	1.118	1.241
All_cause	allAges_Women	7	temp_index_3	1.089	1.044	1.135	1.249	1.106	1.392
All_cause	allAges_Women	7	temp_index_4	1.098	1.067	1.128	1.215	1.130	1.299
All_cause	allAges_Women	10	temp_index_1	1.121	1.083	1.159	1.258	1.133	1.383
All_cause	allAges_Women	10	temp_index_2	1.078	1.039	1.117	1.180	1.109	1.251
All_cause	allAges_Women	10	temp_index_3	1.116	1.055	1.177	1.351	1.146	1.557
All_cause	allAges_Women	10	temp_index_4	1.123	1.083	1.162	1.258	1.149	1.367
All_cause	allAges_Women	15	temp_index_1	1.160	1.107	1.212	1.160	1.016	1.304
All_cause	allAges_Women	15	temp_index_2	1.106	1.056	1.156	1.146	1.059	1.233
All_cause	allAges_Women	15	temp_index_3	1.175	1.098	1.251	1.175	0.952	1.397
All_cause	allAges_Women	15	temp_index_4	1.169	1.117	1.220	1.175	1.047	1.303
All_cause	allAges_Women	20	temp_index_1	1.161	1.094	1.228	1.128	0.963	1.292
All_cause	allAges_Women	20	temp_index_2	1.108	1.047	1.170	1.085	0.988	1.181
All_cause	allAges_Women	20	temp_index_3	1.165	1.105	1.225	1.131	0.872	1.390
All_cause	allAges_Women	20	temp_index_4	1.174	1.112	1.235	1.140	0.992	1.288
All_cause	Age0_14_allSex	3	temp_index_1	1.017	0.992	1.041	1.213	1.068	1.358
All_cause	Age0_14_allSex	3	temp_index_2	1.004	0.992	1.015	1.213	1.112	1.313
All_cause	Age0_14_allSex	3	temp_index_3	1.030	1.000	1.060	1.185	0.991	1.379
All_cause	Age0_14_allSex	3	temp_index_4	1.022	0.999	1.046	1.209	1.076	1.343
All_cause	Age0_14_allSex	5	temp_index_1	1.037	0.999	1.075	1.243	1.074	1.413
All_cause	Age0_14_allSex	5	temp_index_2	1.016	0.990	1.043	1.257	1.138	1.376
All_cause	Age0_14_allSex	5	temp_index_3	1.040	1.004	1.076	1.228	0.999	1.458
All_cause	Age0_14_allSex	5	temp_index_4	1.045	1.009	1.081	1.235	1.080	1.391
All_cause	Age0_14_allSex	7	temp_index_1	1.058	1.008	1.108	1.189	1.003	1.375
All_cause	Age0_14_allSex	7	temp_index_2	1.035	0.999	1.071	1.226	1.093	1.360
All_cause	Age0_14_allSex	7	temp_index_3	1.061	1.013	1.108	1.186	0.927	1.445
All_cause	Age0_14_allSex	7	temp_index_4	1.063	1.018	1.109	1.212	1.036	1.388
All_cause	Age0_14_allSex	10	temp_index_1	1.072	1.008	1.135	1.184	0.960	1.407
All_cause	Age0_14_allSex	10	temp_index_2	1.056	1.009	1.102	1.173	1.024	1.322
All_cause	Age0_14_allSex	10	temp_index_3	1.071	1.006	1.135	1.215	0.889	1.540
All_cause	Age0_14_allSex	10	temp_index_4	1.078	1.019	1.137	1.216	1.005	1.428
All_cause	Age0_14_allSex	15	temp_index_1	1.064	0.987	1.142	1.249	0.962	1.537
All_cause	Age0_14_allSex	15	temp_index_2	1.048	0.989	1.107	1.218	1.028	1.409
All_cause	Age0_14_allSex	15	temp_index_3	1.068	0.987	1.150	1.165	0.794	1.536
All_cause	Age0_14_allSex	15	temp_index_4	1.072	0.997	1.146	1.271	1.006	1.536
All_cause	Age0_14_allSex	20	temp_index_1	1.013	0.942	1.084	1.289	0.987	1.590

All_cause	Age0_14_allSex	20	temp_index_2	1.000	0.958	1.043	1.255	1.039	1.470
All_cause	Age0_14_allSex	20	temp_index_3	0.992	0.910	1.074	1.244	0.882	1.607
All_cause	Age0_14_allSex	20	temp_index_4	1.046	0.970	1.121	1.335	1.015	1.656
All_cause	Age15_45_allSex	3	temp_index_1	1.015	0.998	1.032	1.222	1.114	1.329
All_cause	Age15_45_allSex	3	temp_index_2	1.009	0.998	1.019	1.198	1.123	1.273
All_cause	Age15_45_allSex	3	temp_index_3	1.019	0.997	1.041	1.238	1.092	1.383
All_cause	Age15_45_allSex	3	temp_index_4	1.011	0.999	1.022	1.200	1.104	1.296
All_cause	Age15_45_allSex	5	temp_index_1	1.040	0.999	1.080	1.213	1.092	1.335
All_cause	Age15_45_allSex	5	temp_index_2	1.021	0.998	1.044	1.160	1.082	1.239
All_cause	Age15_45_allSex	5	temp_index_3	1.044	1.002	1.085	1.267	1.094	1.441
All_cause	Age15_45_allSex	5	temp_index_4	1.033	0.995	1.070	1.162	1.060	1.264
All_cause	Age15_45_allSex	7	temp_index_1	1.039	0.994	1.085	1.172	1.034	1.310
All_cause	Age15_45_allSex	7	temp_index_2	1.030	0.989	1.070	1.104	1.021	1.187
All_cause	Age15_45_allSex	7	temp_index_3	1.047	0.999	1.095	1.264	1.053	1.476
All_cause	Age15_45_allSex	7	temp_index_4	1.035	0.989	1.080	1.111	1.000	1.223
All_cause	Age15_45_allSex	10	temp_index_1	1.056	1.004	1.107	1.199	1.036	1.362
All_cause	Age15_45_allSex	10	temp_index_2	1.040	0.991	1.089	1.119	1.019	1.218
All_cause	Age15_45_allSex	10	temp_index_3	1.049	1.000	1.099	1.314	1.048	1.580
All_cause	Age15_45_allSex	10	temp_index_4	1.041	0.987	1.095	1.119	0.988	1.250
All_cause	Age15_45_allSex	15	temp_index_1	1.081	1.015	1.147	1.263	1.033	1.494
All_cause	Age15_45_allSex	15	temp_index_2	1.043	0.983	1.103	1.144	1.018	1.270
All_cause	Age15_45_allSex	15	temp_index_3	1.072	1.000	1.144	1.456	1.041	1.871
All_cause	Age15_45_allSex	15	temp_index_4	1.066	1.005	1.127	1.179	0.994	1.365
All_cause	Age15_45_allSex	20	temp_index_1	1.064	0.984	1.145	1.219	0.941	1.497
All_cause	Age15_45_allSex	20	temp_index_2	1.028	0.968	1.088	1.130	0.964	1.296
All_cause	Age15_45_allSex	20	temp_index_3	1.070	0.980	1.159	1.438	0.911	1.965
All_cause	Age15_45_allSex	20	temp_index_4	1.054	0.975	1.133	1.095	0.886	1.303
All_cause	Age46_65_allSex	3	temp_index_1	1.093	1.055	1.131	1.062	0.983	1.140
All_cause	Age46_65_allSex	3	temp_index_2	1.070	1.032	1.108	1.040	1.002	1.079
All_cause	Age46_65_allSex	3	temp_index_3	1.096	1.041	1.151	1.020	0.959	1.082
All_cause	Age46_65_allSex	3	temp_index_4	1.075	1.041	1.108	1.085	1.011	1.159
All_cause	Age46_65_allSex	5	temp_index_1	1.118	1.074	1.162	1.064	0.973	1.154
All_cause	Age46_65_allSex	5	temp_index_2	1.074	1.030	1.118	1.032	0.989	1.074
All_cause	Age46_65_allSex	5	temp_index_3	1.103	1.040	1.166	1.010	0.941	1.079
All_cause	Age46_65_allSex	5	temp_index_4	1.100	1.062	1.138	1.085	1.001	1.170
All_cause	Age46_65_allSex	7	temp_index_1	1.159	1.102	1.216	1.067	0.969	1.166
All_cause	Age46_65_allSex	7	temp_index_2	1.097	1.045	1.150	1.029	0.981	1.077
All_cause	Age46_65_allSex	7	temp_index_3	1.141	1.066	1.215	1.019	0.938	1.100
All_cause	Age46_65_allSex	7	temp_index_4	1.140	1.093	1.188	1.088	0.993	1.183

All_cause	Age46_65_allSex	10	temp_index_1	1.195	1.127	1.263	1.052	0.935	1.169
All_cause	Age46_65_allSex	10	temp_index_2	1.150	1.074	1.227	0.985	0.932	1.038
All_cause	Age46_65_allSex	10	temp_index_3	1.146	1.060	1.232	0.988	0.895	1.080
All_cause	Age46_65_allSex	10	temp_index_4	1.200	1.137	1.262	1.073	0.962	1.184
All_cause	Age46_65_allSex	15	temp_index_1	1.219	1.134	1.305	1.060	0.910	1.210
All_cause	Age46_65_allSex	15	temp_index_2	1.185	1.087	1.284	0.987	0.919	1.054
All_cause	Age46_65_allSex	15	temp_index_3	1.175	1.066	1.284	0.983	0.865	1.101
All_cause	Age46_65_allSex	15	temp_index_4	1.224	1.143	1.305	1.094	0.949	1.239
All_cause	Age46_65_allSex	20	temp_index_1	1.215	1.115	1.314	1.068	0.881	1.255
All_cause	Age46_65_allSex	20	temp_index_2	1.186	1.071	1.300	0.970	0.889	1.051
All_cause	Age46_65_allSex	20	temp_index_3	1.196	1.073	1.319	0.999	0.834	1.164
All_cause	Age46_65_allSex	20	temp_index_4	1.217	1.122	1.311	1.115	0.924	1.306
All_cause	Age65_allSex	3	temp_index_1	1.090	1.068	1.111	1.206	1.139	1.274
All_cause	Age65_allSex	3	temp_index_2	1.061	1.043	1.080	1.183	1.138	1.228
All_cause	Age65_allSex	3	temp_index_3	1.077	1.052	1.102	1.184	1.091	1.277
All_cause	Age65_allSex	3	temp_index_4	1.085	1.065	1.104	1.207	1.146	1.268
All_cause	Age65_allSex	5	temp_index_1	1.115	1.090	1.141	1.244	1.164	1.324
All_cause	Age65_allSex	5	temp_index_2	1.081	1.058	1.104	1.191	1.139	1.242
All_cause	Age65_allSex	5	temp_index_3	1.109	1.079	1.138	1.276	1.157	1.394
All_cause	Age65_allSex	5	temp_index_4	1.109	1.086	1.132	1.243	1.172	1.314
All_cause	Age65_allSex	7	temp_index_1	1.133	1.102	1.163	1.208	1.120	1.296
All_cause	Age65_allSex	7	temp_index_2	1.087	1.060	1.114	1.167	1.109	1.225
All_cause	Age65_allSex	7	temp_index_3	1.123	1.088	1.157	1.244	1.112	1.376
All_cause	Age65_allSex	7	temp_index_4	1.129	1.100	1.157	1.212	1.133	1.291
All_cause	Age65_allSex	10	temp_index_1	1.158	1.120	1.195	1.171	1.068	1.274
All_cause	Age65_allSex	10	temp_index_2	1.102	1.068	1.135	1.127	1.061	1.193
All_cause	Age65_allSex	10	temp_index_3	1.154	1.111	1.196	1.199	1.042	1.356
All_cause	Age65_allSex	10	temp_index_4	1.155	1.120	1.191	1.181	1.089	1.273
All_cause	Age65_allSex	15	temp_index_1	1.241	1.187	1.294	1.119	0.992	1.245
All_cause	Age65_allSex	15	temp_index_2	1.155	1.107	1.203	1.080	1.001	1.158
All_cause	Age65_allSex	15	temp_index_3	1.236	1.178	1.294	1.127	0.930	1.324
All_cause	Age65_allSex	15	temp_index_4	1.233	1.181	1.286	1.121	1.012	1.229
All_cause	Age65_allSex	20	temp_index_1	1.275	1.207	1.344	1.097	0.947	1.248
All_cause	Age65_allSex	20	temp_index_2	1.164	1.105	1.223	1.039	0.952	1.126
All_cause	Age65_allSex	20	temp_index_3	1.257	1.185	1.330	1.062	0.830	1.294
All_cause	Age65_allSex	20	temp_index_4	1.284	1.219	1.349	1.117	0.985	1.250
All_cause	Age0_14_Men	3	temp_index_1	1.035	0.965	1.104	1.102	0.936	1.269
All_cause	Age0_14_Men	3	temp_index_2	0.997	0.990	1.005	1.171	1.059	1.283
All_cause	Age0_14_Men	3	temp_index_3	1.033	0.959	1.107	1.108	0.868	1.348

All_cause	Age0_14_Men	3	temp_index_4	1.030	0.994	1.066	1.168	0.998	1.338
All_cause	Age0_14_Men	5	temp_index_1	1.024	0.980	1.067	1.171	0.962	1.379
All_cause	Age0_14_Men	5	temp_index_2	1.005	0.975	1.035	1.184	1.054	1.313
All_cause	Age0_14_Men	5	temp_index_3	0.999	0.950	1.048	1.145	0.926	1.364
All_cause	Age0_14_Men	5	temp_index_4	1.034	0.991	1.078	1.189	0.991	1.387
All_cause	Age0_14_Men	7	temp_index_1	1.022	0.947	1.097	1.121	0.947	1.296
All_cause	Age0_14_Men	7	temp_index_2	1.022	0.970	1.075	1.159	1.016	1.301
All_cause	Age0_14_Men	7	temp_index_3	0.988	0.848	1.128	1.090	0.871	1.310
All_cause	Age0_14_Men	7	temp_index_4	1.033	0.960	1.105	1.085	0.933	1.237
All_cause	Age0_14_Men	10	temp_index_1	0.976	0.821	1.132	1.070	0.874	1.265
All_cause	Age0_14_Men	10	temp_index_2	1.024	0.886	1.162	1.067	0.922	1.212
All_cause	Age0_14_Men	10	temp_index_3	0.985	0.819	1.151	1.122	0.844	1.399
All_cause	Age0_14_Men	10	temp_index_4	0.985	0.838	1.131	1.018	0.849	1.186
All_cause	Age0_14_Men	15	temp_index_1	0.988	0.962	1.014	1.231	0.924	1.538
All_cause	Age0_14_Men	15	temp_index_2	0.989	0.972	1.006	1.114	0.921	1.307
All_cause	Age0_14_Men	15	temp_index_3	0.971	0.945	0.997	1.333	0.842	1.824
All_cause	Age0_14_Men	15	temp_index_4	0.995	0.927	1.063	1.164	0.897	1.431
All_cause	Age0_14_Men	20	temp_index_1	0.983	0.951	1.015	1.287	0.861	1.712
All_cause	Age0_14_Men	20	temp_index_2	0.989	0.969	1.010	1.179	0.916	1.442
All_cause	Age0_14_Men	20	temp_index_3	0.970	0.958	0.983	1.328	0.706	1.949
All_cause	Age0_14_Men	20	temp_index_4	0.981	0.957	1.006	1.217	0.829	1.604
All_cause	Age15_45_Men	3	temp_index_1	1.005	0.986	1.025	1.221	1.095	1.348
All_cause	Age15_45_Men	3	temp_index_2	1.013	1.001	1.025	1.194	1.106	1.282
All_cause	Age15_45_Men	3	temp_index_3	1.011	0.986	1.035	1.273	1.091	1.455
All_cause	Age15_45_Men	3	temp_index_4	1.008	0.995	1.020	1.184	1.072	1.296
All_cause	Age15_45_Men	5	temp_index_1	1.014	0.986	1.041	1.267	1.113	1.421
All_cause	Age15_45_Men	5	temp_index_2	1.020	0.998	1.042	1.184	1.086	1.282
All_cause	Age15_45_Men	5	temp_index_3	1.021	0.992	1.049	1.361	1.127	1.594
All_cause	Age15_45_Men	5	temp_index_4	1.001	0.978	1.024	1.184	1.057	1.310
All_cause	Age15_45_Men	7	temp_index_1	1.041	0.989	1.092	1.232	1.057	1.408
All_cause	Age15_45_Men	7	temp_index_2	1.032	0.988	1.076	1.116	1.011	1.220
All_cause	Age15_45_Men	7	temp_index_3	1.044	0.993	1.096	1.363	1.081	1.645
All_cause	Age15_45_Men	7	temp_index_4	1.023	0.977	1.069	1.129	0.989	1.268
All_cause	Age15_45_Men	10	temp_index_1	1.053	0.991	1.115	1.247	1.041	1.452
All_cause	Age15_45_Men	10	temp_index_2	1.051	0.993	1.108	1.126	1.002	1.249
All_cause	Age15_45_Men	10	temp_index_3	1.051	0.990	1.112	1.406	1.062	1.749
All_cause	Age15_45_Men	10	temp_index_4	1.037	0.981	1.092	1.130	0.966	1.294
All_cause	Age15_45_Men	15	temp_index_1	1.078	0.998	1.157	1.272	0.993	1.550
All_cause	Age15_45_Men	15	temp_index_2	1.051	0.980	1.123	1.103	0.946	1.260

All_cause	Age15_45_Men	15	temp_index_3	1.080	1.000	1.159	1.508	0.986	2.030
All_cause	Age15_45_Men	15	temp_index_4	1.051	0.975	1.127	1.123	0.918	1.327
All_cause	Age15_45_Men	20	temp_index_1	1.088	0.989	1.186	1.126	0.819	1.433
All_cause	Age15_45_Men	20	temp_index_2	1.050	0.956	1.144	1.008	0.845	1.171
All_cause	Age15_45_Men	20	temp_index_3	1.083	0.988	1.178	1.300	0.726	1.873
All_cause	Age15_45_Men	20	temp_index_4	1.058	0.960	1.156	1.006	0.781	1.231
All_cause	Age46_65_Men	3	temp_index_1	1.087	1.039	1.135	1.071	0.966	1.175
All_cause	Age46_65_Men	3	temp_index_2	1.055	1.013	1.097	1.036	0.977	1.096
All_cause	Age46_65_Men	3	temp_index_3	1.108	1.053	1.164	1.026	0.897	1.154
All_cause	Age46_65_Men	3	temp_index_4	1.072	1.030	1.115	1.088	0.992	1.184
All_cause	Age46_65_Men	5	temp_index_1	1.115	1.056	1.175	1.065	0.951	1.180
All_cause	Age46_65_Men	5	temp_index_2	1.071	1.019	1.124	1.017	0.959	1.075
All_cause	Age46_65_Men	5	temp_index_3	1.139	1.071	1.207	1.016	0.877	1.155
All_cause	Age46_65_Men	5	temp_index_4	1.098	1.047	1.150	1.083	0.977	1.190
All_cause	Age46_65_Men	7	temp_index_1	1.160	1.089	1.232	1.094	0.958	1.230
All_cause	Age46_65_Men	7	temp_index_2	1.101	1.024	1.179	1.008	0.949	1.067
All_cause	Age46_65_Men	7	temp_index_3	1.186	1.105	1.266	1.054	0.883	1.225
All_cause	Age46_65_Men	7	temp_index_4	1.140	1.079	1.200	1.114	0.984	1.243
All_cause	Age46_65_Men	10	temp_index_1	1.200	1.113	1.287	1.046	0.896	1.195
All_cause	Age46_65_Men	10	temp_index_2	1.158	1.058	1.258	0.971	0.905	1.037
All_cause	Age46_65_Men	10	temp_index_3	1.180	1.067	1.292	0.993	0.874	1.113
All_cause	Age46_65_Men	10	temp_index_4	1.204	1.124	1.285	1.081	0.936	1.227
All_cause	Age46_65_Men	15	temp_index_1	1.216	1.074	1.358	1.006	0.877	1.134
All_cause	Age46_65_Men	15	temp_index_2	1.210	1.077	1.342	0.965	0.882	1.047
All_cause	Age46_65_Men	15	temp_index_3	1.242	1.095	1.389	0.987	0.832	1.142
All_cause	Age46_65_Men	15	temp_index_4	1.228	1.120	1.337	1.095	0.914	1.276
All_cause	Age46_65_Men	20	temp_index_1	1.248	1.112	1.383	1.094	0.851	1.338
All_cause	Age46_65_Men	20	temp_index_2	1.212	1.058	1.367	0.980	0.876	1.084
All_cause	Age46_65_Men	20	temp_index_3	1.294	1.119	1.469	1.040	0.836	1.243
All_cause	Age46_65_Men	20	temp_index_4	1.245	1.120	1.370	1.189	0.922	1.456
All_cause	Age65_Men	3	temp_index_1	1.116	1.084	1.147	1.132	1.046	1.217
All_cause	Age65_Men	3	temp_index_2	1.075	1.048	1.103	1.120	1.062	1.177
All_cause	Age65_Men	3	temp_index_3	1.110	1.074	1.146	1.087	0.972	1.202
All_cause	Age65_Men	3	temp_index_4	1.091	1.062	1.121	1.124	1.048	1.200
All_cause	Age65_Men	5	temp_index_1	1.142	1.103	1.181	1.165	1.063	1.268
All_cause	Age65_Men	5	temp_index_2	1.098	1.065	1.131	1.126	1.060	1.192
All_cause	Age65_Men	5	temp_index_3	1.146	1.103	1.188	1.164	1.018	1.311
All_cause	Age65_Men	5	temp_index_4	1.120	1.085	1.155	1.165	1.074	1.255
All_cause	Age65_Men	7	temp_index_1	1.172	1.127	1.218	1.143	1.029	1.258

All_cause	Age65_Men	7	temp_index_2	1.104	1.066	1.142	1.113	1.037	1.189
All_cause	Age65_Men	7	temp_index_3	1.162	1.111	1.213	1.144	0.977	1.311
All_cause	Age65_Men	7	temp_index_4	1.150	1.108	1.191	1.129	1.028	1.231
All_cause	Age65_Men	10	temp_index_1	1.207	1.150	1.263	1.084	0.958	1.209
All_cause	Age65_Men	10	temp_index_2	1.130	1.081	1.180	1.067	0.984	1.150
All_cause	Age65_Men	10	temp_index_3	1.190	1.129	1.251	1.089	0.899	1.279
All_cause	Age65_Men	10	temp_index_4	1.199	1.147	1.250	1.090	0.975	1.205
All_cause	Age65_Men	15	temp_index_1	1.308	1.230	1.386	1.095	0.925	1.265
All_cause	Age65_Men	15	temp_index_2	1.201	1.132	1.270	1.051	0.947	1.155
All_cause	Age65_Men	15	temp_index_3	1.281	1.199	1.363	1.115	0.845	1.386
All_cause	Age65_Men	15	temp_index_4	1.302	1.228	1.376	1.094	0.947	1.241
All_cause	Age65_Men	20	temp_index_1	1.336	1.238	1.435	1.124	0.902	1.346
All_cause	Age65_Men	20	temp_index_2	1.212	1.128	1.297	1.063	0.939	1.188
All_cause	Age65_Men	20	temp_index_3	1.301	1.197	1.405	1.087	0.729	1.444
All_cause	Age65_Men	20	temp_index_4	1.354	1.261	1.446	1.134	0.946	1.322
All_cause	Age0_14_Women	3	temp_index_1	1.022	0.989	1.055	1.334	1.078	1.590
All_cause	Age0_14_Women	3	temp_index_2	1.021	1.003	1.039	1.372	1.185	1.560
All_cause	Age0_14_Women	3	temp_index_3	1.034	0.991	1.078	1.285	0.942	1.628
All_cause	Age0_14_Women	3	temp_index_4	1.026	0.996	1.056	1.297	1.070	1.523
All_cause	Age0_14_Women	5	temp_index_1	1.057	0.992	1.121	1.355	1.056	1.654
All_cause	Age0_14_Women	5	temp_index_2	1.030	0.994	1.066	1.442	1.217	1.667
All_cause	Age0_14_Women	5	temp_index_3	1.059	0.998	1.120	1.325	0.908	1.742
All_cause	Age0_14_Women	5	temp_index_4	1.063	1.004	1.122	1.304	1.043	1.564
All_cause	Age0_14_Women	7	temp_index_1	1.074	0.997	1.151	1.473	1.065	1.881
All_cause	Age0_14_Women	7	temp_index_2	1.044	0.989	1.098	1.488	1.222	1.754
All_cause	Age0_14_Women	7	temp_index_3	1.080	1.005	1.155	1.539	0.920	2.158
All_cause	Age0_14_Women	7	temp_index_4	1.073	1.002	1.143	1.446	1.092	1.801
All_cause	Age0_14_Women	10	temp_index_1	1.127	1.022	1.232	1.706	1.100	2.313
All_cause	Age0_14_Women	10	temp_index_2	1.106	1.025	1.188	1.606	1.269	1.943
All_cause	Age0_14_Women	10	temp_index_3	1.137	1.030	1.244	0.807	0.536	1.079
All_cause	Age0_14_Women	10	temp_index_4	1.131	1.034	1.227	1.627	1.122	2.133
All_cause	Age0_14_Women	15	temp_index_1	1.122	0.986	1.259	1.267	0.743	1.791
All_cause	Age0_14_Women	15	temp_index_2	1.113	1.002	1.224	1.573	1.168	1.978
All_cause	Age0_14_Women	15	temp_index_3	1.001	0.834	1.169	0.811	0.385	1.238
All_cause	Age0_14_Women	15	temp_index_4	1.118	0.988	1.248	1.350	0.858	1.842
All_cause	Age0_14_Women	20	temp_index_1	1.094	0.948	1.239	1.638	0.807	2.469
All_cause	Age0_14_Women	20	temp_index_2	1.092	0.974	1.209	1.672	1.133	2.212
All_cause	Age0_14_Women	20	temp_index_3	1.045	0.884	1.206	1.033	0.498	1.568
All_cause	Age0_14_Women	20	temp_index_4	1.103	0.971	1.234	1.713	0.949	2.478

All_cause	Age15_45_Women	3	temp_index_1	1.019	0.978	1.059	1.242	1.028	1.455
All_cause	Age15_45_Women	3	temp_index_2	1.013	0.993	1.034	1.246	1.098	1.393
All_cause	Age15_45_Women	3	temp_index_3	1.020	0.971	1.070	1.089	0.951	1.226
All_cause	Age15_45_Women	3	temp_index_4	1.030	1.002	1.057	1.298	1.092	1.505
All_cause	Age15_45_Women	5	temp_index_1	1.106	1.016	1.195	1.131	0.929	1.333
All_cause	Age15_45_Women	5	temp_index_2	1.065	0.984	1.145	1.121	0.992	1.250
All_cause	Age15_45_Women	5	temp_index_3	1.078	0.956	1.199	1.063	0.914	1.213
All_cause	Age15_45_Women	5	temp_index_4	1.110	1.026	1.193	1.157	0.970	1.345
All_cause	Age15_45_Women	7	temp_index_1	1.061	0.929	1.193	1.064	0.897	1.232
All_cause	Age15_45_Women	7	temp_index_2	1.046	0.938	1.155	1.109	0.971	1.247
All_cause	Age15_45_Women	7	temp_index_3	1.066	0.937	1.196	1.009	0.846	1.173
All_cause	Age15_45_Women	7	temp_index_4	1.083	0.984	1.182	1.110	0.903	1.317
All_cause	Age15_45_Women	10	temp_index_1	1.064	1.013	1.116	1.089	0.904	1.274
All_cause	Age15_45_Women	10	temp_index_2	1.031	0.998	1.064	1.127	0.974	1.279
All_cause	Age15_45_Women	10	temp_index_3	1.037	0.961	1.112	1.021	0.820	1.221
All_cause	Age15_45_Women	10	temp_index_4	1.054	0.921	1.187	1.105	0.931	1.280
All_cause	Age15_45_Women	15	temp_index_1	1.104	0.915	1.292	1.066	0.802	1.330
All_cause	Age15_45_Women	15	temp_index_2	1.062	0.983	1.142	1.032	0.871	1.192
All_cause	Age15_45_Women	15	temp_index_3	1.117	0.998	1.236	1.038	0.748	1.328
All_cause	Age15_45_Women	15	temp_index_4	1.091	0.921	1.261	1.019	0.833	1.204
All_cause	Age15_45_Women	20	temp_index_1	1.055	0.958	1.152	1.308	0.660	1.957
All_cause	Age15_45_Women	20	temp_index_2	1.021	0.964	1.078	1.276	0.900	1.652
All_cause	Age15_45_Women	20	temp_index_3	1.071	0.944	1.198	1.388	0.083	2.693
All_cause	Age15_45_Women	20	temp_index_4	1.045	0.961	1.129	1.231	0.726	1.735
All_cause	Age46_65_Women	3	temp_index_1	1.104	1.023	1.186	1.094	1.003	1.186
All_cause	Age46_65_Women	3	temp_index_2	1.095	1.032	1.157	1.064	1.000	1.128
All_cause	Age46_65_Women	3	temp_index_3	1.085	0.995	1.176	1.069	0.963	1.175
All_cause	Age46_65_Women	3	temp_index_4	1.109	1.038	1.180	1.077	0.998	1.155
All_cause	Age46_65_Women	5	temp_index_1	1.123	1.038	1.209	1.084	0.983	1.185
All_cause	Age46_65_Women	5	temp_index_2	1.088	1.024	1.153	1.059	0.987	1.130
All_cause	Age46_65_Women	5	temp_index_3	1.101	1.021	1.180	1.035	0.919	1.152
All_cause	Age46_65_Women	5	temp_index_4	1.124	1.046	1.202	1.077	0.988	1.165
All_cause	Age46_65_Women	7	temp_index_1	1.127	1.008	1.246	1.068	0.955	1.182
All_cause	Age46_65_Women	7	temp_index_2	1.104	1.017	1.192	1.052	0.970	1.133
All_cause	Age46_65_Women	7	temp_index_3	1.108	0.989	1.227	1.035	0.900	1.170
All_cause	Age46_65_Women	7	temp_index_4	1.138	1.041	1.235	1.067	0.965	1.169
All_cause	Age46_65_Women	10	temp_index_1	1.186	1.075	1.297	1.042	0.866	1.219
All_cause	Age46_65_Women	10	temp_index_2	1.112	1.009	1.214	0.987	0.898	1.076
All_cause	Age46_65_Women	10	temp_index_3	1.093	0.959	1.227	0.975	0.825	1.126

All_cause	Age46_65_Women	10	temp_index_4	1.152	1.037	1.267	1.022	0.909	1.134
All_cause	Age46_65_Women	15	temp_index_1	1.214	1.080	1.347	1.093	0.814	1.372
All_cause	Age46_65_Women	15	temp_index_2	1.182	1.053	1.311	1.037	0.878	1.196
All_cause	Age46_65_Women	15	temp_index_3	1.180	1.048	1.312	1.180	0.690	1.671
All_cause	Age46_65_Women	15	temp_index_4	1.236	1.110	1.362	1.090	0.837	1.343
All_cause	Age46_65_Women	20	temp_index_1	1.181	1.032	1.329	1.031	0.714	1.348
All_cause	Age46_65_Women	20	temp_index_2	1.171	1.021	1.320	0.980	0.812	1.148
All_cause	Age46_65_Women	20	temp_index_3	1.181	1.057	1.305	1.127	0.540	1.715
All_cause	Age46_65_Women	20	temp_index_4	1.149	0.950	1.347	1.055	0.821	1.290
All_cause	Age65_Women	3	temp_index_1	1.067	1.040	1.095	1.299	1.188	1.409
All_cause	Age65_Women	3	temp_index_2	1.047	1.024	1.071	1.259	1.187	1.331
All_cause	Age65_Women	3	temp_index_3	1.042	1.010	1.074	1.305	1.147	1.463
All_cause	Age65_Women	3	temp_index_4	1.067	1.044	1.089	1.290	1.192	1.388
All_cause	Age65_Women	5	temp_index_1	1.088	1.054	1.122	1.333	1.204	1.461
All_cause	Age65_Women	5	temp_index_2	1.063	1.033	1.094	1.267	1.185	1.349
All_cause	Age65_Women	5	temp_index_3	1.070	1.032	1.109	1.401	1.201	1.602
All_cause	Age65_Women	5	temp_index_4	1.087	1.057	1.118	1.314	1.203	1.425
All_cause	Age65_Women	7	temp_index_1	1.096	1.054	1.137	1.274	1.136	1.412
All_cause	Age65_Women	7	temp_index_2	1.067	1.028	1.105	1.226	1.136	1.315
All_cause	Age65_Women	7	temp_index_3	1.068	1.026	1.110	1.332	1.113	1.551
All_cause	Age65_Women	7	temp_index_4	1.094	1.054	1.134	1.271	1.150	1.392
All_cause	Age65_Women	10	temp_index_1	1.114	1.064	1.163	1.296	1.117	1.476
All_cause	Age65_Women	10	temp_index_2	1.073	1.027	1.118	1.204	1.097	1.310
All_cause	Age65_Women	10	temp_index_3	1.111	1.052	1.170	1.341	1.062	1.620
All_cause	Age65_Women	10	temp_index_4	1.107	1.054	1.161	1.292	1.139	1.445
All_cause	Age65_Women	15	temp_index_1	1.163	1.090	1.235	1.137	0.942	1.332
All_cause	Age65_Women	15	temp_index_2	1.102	1.036	1.167	1.108	0.988	1.229
All_cause	Age65_Women	15	temp_index_3	1.196	1.092	1.301	1.145	0.829	1.462
All_cause	Age65_Women	15	temp_index_4	1.164	1.089	1.239	1.151	0.984	1.318
All_cause	Age65_Women	20	temp_index_1	1.199	1.104	1.294	1.057	0.849	1.265
All_cause	Age65_Women	20	temp_index_2	1.126	1.056	1.197	1.010	0.872	1.148
All_cause	Age65_Women	20	temp_index_3	1.208	1.114	1.302	0.997	0.691	1.302
All_cause	Age65_Women	20	temp_index_4	1.207	1.117	1.297	1.093	0.898	1.287
